# Folate receptor overexpression induces toxicity in a diet-dependent manner in *C. elegans*

**DOI:** 10.1038/s41598-024-51700-9

**Published:** 2024-01-11

**Authors:** Bideep Shrestha, Milla Tallila, Olli Matilainen

**Affiliations:** The Molecular and Integrative Biosciences Research Programme, Faculty of Biological and Environmental Sciences, University of Helsinki, Helsinki, Finland

**Keywords:** Ageing, Disease model, Experimental organisms

## Abstract

Folate receptor (FR) alpha (FOLR1) and beta (FOLR2) are membrane-anchored folate transporters that are expressed at low levels in normal tissues, while their expression is strongly increased in several cancers. Intriguingly, although the function of these receptors in, for example, development and cancer has been studied intensively, their role in aging is still unknown. To address this, we utilized *Caenorhabditis elegans*, in which FOLR-1 is the sole ortholog of folate receptors. We found that the loss of FOLR-1 does not affect reproduction, physical condition, proteostasis or lifespan, indicating that it is not required for folate transport to maintain health. Interestingly, we found that FOLR-1 is detectably expressed only in uterine-vulval cells, and that the histone-binding protein LIN-53 inhibits its expression in other tissues. Furthermore, whereas knockdown of *lin-53* is known to shorten lifespan, we found that the loss of FOLR-1 partially rescues this phenotype, suggesting that elevated *folr-1* expression is detrimental for health. Indeed, our data demonstrate that overexpression of *folr-1* is toxic, and that this phenotype is dependent on diet. Altogether, this work could serve as a basis for further studies to elucidate the organismal effects of abnormal FR expression in diseases such as cancer.

## Introduction

Folate, also known as vitamin B_9_, is a cofactor in one-carbon metabolism (OCM), which supports processes such as purine, thymidine and methionine biosynthesis, homeostasis of glycine and serine, epigenetic maintenance, and redox defense^1^. Eukaryotic organisms such as nematodes, flies and mammals cannot synthesize folates, and therefore, they must obtain them from the diet or through biosynthesis within the gut microbiota. Due to its central role in OCM, folate deficiency contributes to multiple pathologies including cancer, cardiovascular disease, and developmental anomalies such as neural tube defects^2^. In addition to their role in preventing diseases and promoting development, folate and the associated OCM regulate aging in multiple organisms^3^. As an example from *C. elegans*, it has been shown that altered function of OCM is a common signature of long-lived *C. elegans* strains, and that OCM downregulation triggers lifespan-extending methionine restriction^4^. Moreover, metformin, a drug to treat type-2-diabetes, inhibits microbial folate and methionine metabolism, which leads to extended lifespan through methionine restriction^5^. Furthermore, it has been shown that the inhibition of microbial folate synthesis leads to extended longevity^6,7^, which further strengthens the connection between folate, microbiome, and aging. Hence, extensive research on folate and its metabolism reveals its crucial role in various cellular processes.

In humans, the majority of folate is transported to cells by three transporters: the proton-coupled folate transporter (PCFT), the reduced folate carrier (RFC) and folate receptors (transport folates into cells via an endocytic mechanism). PCFT functions mainly in the upper gastrointestinal tract^8^, whereas RFC is ubiquitously expressed^9^. Although RFC is the major factor transporting folate to tissues, it binds folates with relatively low affinity (K_m_ = 1–10 μM)^10^. In contrast to RFC, folate receptors (FRs, include three folate-binding isoforms: alpha (FOLR1), beta (FOLR2) and gamma (FOLR3)) bind folates with high affinity^11^. For example, FOLR1 binds synthetic folic acid (Kd: < 1 nM) and 5-methyltetrahydrofolate (5-MTHF) (Kd: 1–10 nM) efficiently at low physiologic concentrations^12^. Unlike RFC, folate receptors show a restricted expression profile. The expression of constitutively secreted FOLR3 can be detected in normal and leukemic hematopoietic tissues, whereas the membrane-bound FOLR2 can be found in placenta and hematopoietic cells^13^. The membrane-bound FOLR1, the most studied and widely expressed FR, is expressed in the choroid plexus, lung, thyroid, and kidney^13–16^. Notably, although FRs show low expression in normal cells, their expression is strongly increased in multiple cancers^14,15,17–21^. Even though the expression profiles of FRs’ have been well documented, the factors regulating their expression are still unknown.

*C. elegans* has two identified folate transporters, FOLT-1 (homolog of RFC)^22,23^ and FOLR-1 (homolog of FR)^24^. *folt-1* is ubiquitously expressed with the pharynx and intestine displaying the strongest expression^22^. The loss of FOLT-1 causes germline and somatic defects, as well as a shortened lifespan^23^, thus demonstrating the importance of this folate transporter for *C. elegans* development and normal aging. Regarding FOLR-1, it has been shown that bacterial folates stimulate germ cell proliferation through this receptor^24^. This is an interesting observation because folates, which have a role as vitamins, do not stimulate germ cell proliferation, thereby indicating that FOLR-1 is able to regulate germ cell number independently of OCM^24^. The OCM-independent role of FR is not limited to *C. elegans*, as multiple studies have demonstrated that FOLR1 is a regulator of JAK–STAT3 and ERK1/2 signaling pathways in mammalian systems^17^. Moreover, it has been shown that FOLR1 is able to translocate into the nucleus and function as a transcription factor^25–27^, providing further evidence for non-canonical functions of FOLR1. As mentioned earlier, folate metabolism and OCM are important regulators of aging^4–7,23^. Although FR can function both as a part of OCM and independently of it^17^, it is not known whether FR modulates aging. To address this, we utilized *C. elegans* to elucidate how this receptor affects health and longevity.

## Results

### FOLR1 is detectably expressed only in uterine-vulval cells and its expression in other tissues is inhibited by the histone-binding protein LIN-53

**Figure 1:**
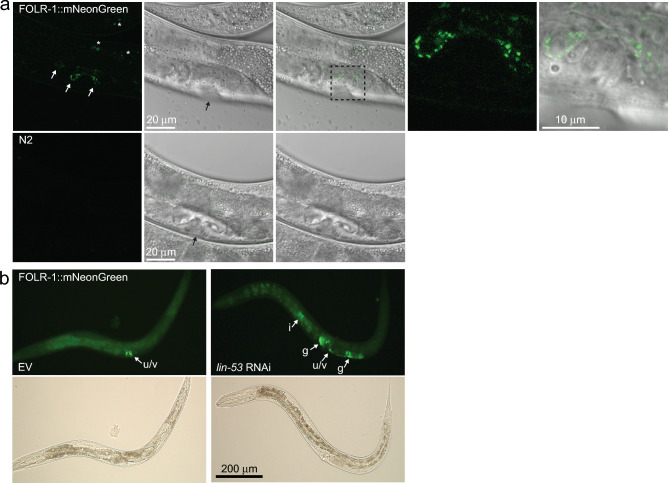
FOLR-1 expression profile. (**a**) Confocal images of FOLR-1::mNeonGreen fusion protein-expressing animals and N2 (wild-type) at the late L4 stage when the vulval lumen (black arrow) begins to narrow. White arrows indicate FOLR-1::mNeonGreen localization. N2 is used to demonstrate that the fluorescence signal from the uterine-vulval cells is not background noise. Autofluorescence from gut granules is marked with asterisks. The dashed square marks the magnified area shown on the right. All FOLR-1::mNeonGreen confocal images shown in this manuscript were taken with the same settings during the same imaging session, facilitating the comparison of the images. (**b**) Images of the late L4 stage FOLR-1::mNeonGreen animals treated with EV (empty vector) or *lin-53* RNAi. Arrows indicate FOLR-1::mNeonGreen expression (u/v, uterine/vulval region; i, intestine; g, gonad). The experiment was repeated three times with similar results.

As the first step, we examined how the FOLR-1 protein is expressed in *C. elegans*. For this purpose, we used a strain in which the endogenous FOLR-1 is tagged with the fluorescent mNeonGreen protein by CRISPR/Cas9-mediated genome editing. Interestingly, when imaging the FOLR-1::mNeonGreen-strain at the L4 larval stage, we found that FOLR-1 can only be detected in a few cells close to the vulva (Fig. 1a and Fig. S1). Notably, the single-cell resolution atlas of gene activity of young adult *C. elegans* (WormSeq application)^28^ revealed that *folr-1* mRNA is expressed at least in uterine-vulval cells, dorsal uterine cell and the RIS neuron (Figs. S2 and S3). By comparing the expression profile of FOLR-1::mNeonGreen (Fig. 1a and Fig. S1) with the structural anatomy of the *C. elegans* reproductive system^29^, we found that FOLR-1 can be detected at least in uterine-vulval cells. There are three types of uterine-vulval cells (uv-1–3), which form a multilayered set of flaps that bind the ventral uterus to the dorsalmost ring of the vulva^29^. The expression in uterine-vulval cells becomes visible at the late L4 stage when the vulval lumen begins to narrow (Fig. 1a). The FOLR-1::mNeonGreen signal reaches its strongest level when the vulval lumen closes at the transition from the late L4 stage to the young adult stage (Fig. S1). On the contrary to the late L4 stage, the FOLR-1::mNeonGreen signal cannot be detected at mid-L4 stage (Fig. S1). Furthermore, the FOLR-1::mNeonGreen signal is gone in most of the gravid day 1 adults (Fig. S1), demonstrating that FOLR-1 is expressed in a developmental stage-specific manner. Together, these assays (single-cell RNA-seq^28^ and imaging of the FOLR-1::mNeonGreen-expressing strain) demonstrate that FOLR-1 can only be detected in a few cell types. However, it is noteworthy that FOLR-1 can also be expressed in other tissues (such as the RIS neuron), but the sensitivity of FOLR-1::mNeonGreen may not be sufficient to detect it at low levels.

Due to its restricted expression pattern, it is possible that there are specific factors that inhibit FOLR-1 expression in other tissues. LIN-53, the homolog of the mammalian histone-binding protein RbAp48, has been shown to repress the transcription of genes determining vulval cell fates^30^. Furthermore, it has been demonstrated that germ cells acquire the ability to be reprogrammed into distinct neuron types upon the loss of *lin-53*^31^. Therefore, we asked whether LIN-53 regulates FOLR-1 expression. Strikingly, we found that *lin-53* RNAi induces ectopic FOLR-1 expression in the intestine and gonad (Fig. 1b), hence demonstrating that LIN-53 controls the restricted expression pattern of FOLR-1.

### The loss of FOLR-1 does not affect lifespan, activity, progeny number, or proteotoxicity

**Figure 2:**
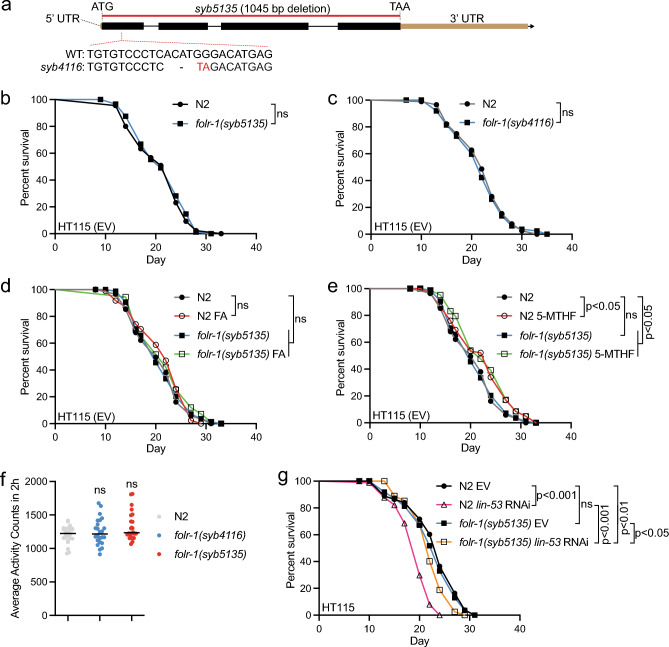
Loss of FOLR-1 does not affect lifespan, activity, or brood size, but partially rescues the shortened lifespan upon *lin-53* RNAi (**a**) Schematic presentation of *folr-1* gene with mutations generated for this study. (**b**) Lifespan of *folr-1(syb4116)* and (**c**) *folr-1(syb5135)* mutants on HT115 (carrying empty vector, EV) compared to N2. Data in both (**b**) and (**c**) are representative of two independent experiments. (**d**–**e**) Lifespan of *folr-1(syb5135)* mutant and N2 on plates supplemented with (**d**) 10 μM folic acid (FA) or (**e**) 100 nM 5-methyltetrahydrofolate (5-MTHF). Data in both (**d**) and (**e**) are representative of at least two independent experiments. (**f**) Activity of day 4 adult N2, *folr-1(syb4116)* and *folr-1(syb5135)* mutants measured with wMicroTracker. Each dot represents a group of 10 animals (n = 240 animals per condition). Data are combined from two independent experiments. Statistical significances were calculated with one-way ANOVA with Tukey’s test. (**g**) Lifespan of N2 and *folr-1(syb5135)* mutants on *lin-53* RNAi. Data are representative of two independent experiments. Statistical calculations for lifespan experiments were performed using the Cox-proportional hazard regression analysis. Lifespan statistics are reported in Supplementary Information file 1, Table S1.

Next, we asked whether the loss of FOLR-1 influences animal physiology. For this purpose, we utilized two separate loss-of-function *folr-1* mutant strains generated by CRISPR/Cas9-mediated genome editing. The *folr-1(syb4116)* allele has a premature stop codon after 20 amino acids, whereas the *folr-1(syb5135)* allele is a complete deletion of the *folr-1* gene (from start to stop codon) (Fig. 2a). Initially, to assess the impact of FOLR-1 depletion on physiology, we examined its effects on lifespan. For these experiments, we used *E. coli* HT115 (carrying an empty RNAi vector, EV). Since HT115 is commonly used in RNAi experiments, we chose these bacteria as it allows the subsequent exploration of potential lifespan modulators through knockdowns. We found that both *folr-1* mutant alleles have a normal lifespan (Fig. 2b and c). Additionally, the loss of *folr-1* does not affect survival in higher temperature (Fig. S4a). As the next step we investigated whether folate supplementation extends lifespan, and whether FOLR-1 is required for this process. Folic acid (FA) is a synthetic form of folate that is widely used as a food supplement. Interestingly, mammalian FOLR1 has a 14-fold higher affinity for FA compared to the naturally occurring 5-methyltetrahydrofolate (5-MTHF)^32^, whereas *C. elegans* FOLR-1 does not promote germ cell proliferation in response to FA^24^. This suggests that the role of the folate receptor in FA uptake varies between species. Nevertheless, it has been published that 10 μM and 25 μM FA solutions spread on agar plates with *E. coli* OP50 as a food source extend *C. elegans* lifespan^33^. In our experiment we supplemented NGM agar with FA at final concentration of 10 μM and used *E. coli* HT115 as a food source. In these experiments, FA did not affect the lifespan of N2 or *folr-1(syb5135)* mutants (Fig. 2d). Notably, in our experiments FA did not affect N2 lifespan even when used at a final concentration of 250 μM (Fig. S4b). Next, we examined the lifespan effects of 5-MTHF in N2 and *folr-1(syb5135)* animals. 5-MTHF is an intermediate in OCM, and like FA, it is also used as a food supplement. Interestingly, since decreased OCM activity extends lifespan, 5-MTHF shortens the lifespan of OCM-deficient animals (and long-lived mutants) when used at a low concentration (10 nM)^4^. Surprisingly, we found that 5-MTHF used at a final concentration of 100 nM in NGM agar extends the lifespan of both N2 and *folr-1(syb5135)* animals (Fig. 2e). Regarding humans, in healthy adults, plasma and red cell 5-MTHF levels are in the range of 6.6–39.9 nM and 223–1041 nM, respectively^34^. Therefore, our data demonstrate that 5-MTHF promotes longevity at a concentration that is in the middle of these two reported physiological 5-MTHF levels. Furthermore, our data demonstrate that FOLR-1 is not required for the longevity-inducing effect of this folate species.

In addition to its role in longevity, we also investigated whether the loss of FOLR-1 affects activity, which is often analyzed as it reflects physical condition. Firstly, we measured the activity of *folr-1* mutants at day 4 of adulthood. In line with the observations from the lifespan assays (Fig. 2b and c), *folr-1* mutants do not show a difference in activity compared to N2 animals (Fig. 2f). Secondly, since the loss of FOLT-1 causes sterility^23^, we tested whether FOLR-1 affects reproduction and found that the loss of this receptor does not affect the brood size (Fig. S4c). These data support the earlier study reporting that *folr-1* RNAi does not affect the number of eggs laid^24^.

Folate deficiency increases the risk of Alzheimer’s disease (AD), while sufficient folate intake protects from this disorder^35^. Since the toxicity of amyloid beta (Aβ) oligomers is a central part of AD, we asked whether *folr-1* mutation modulates Aβ toxicity. For this purpose, we crossed *folr-1(syb4116)* mutant with GMC101, a strain expressing Aβ_1–42_ peptide in body-wall muscle cells^36^. Western blot experiment showed that *folr-1(syb4116)* mutation does not affect Aβ accumulation (Fig. S4d). Furthermore, *folr-1* depletion does not affect the activity of GMC101 animals (Fig. S4e), demonstrating that FOLR-1 is not required to maintain proteostasis. Interestingly, in contrast to the loss of FOLR-1, we found that *folt-1* RNAi increases the accumulation of Aβ oligomers (Fig. S4f), which supports previous findings reporting that folate plays a protective role in AD^35^. As the loss of FOLR-1 and *folt-1* RNAi exhibit differential effects on Aβ accumulation, these data provide additional evidence that FOLR-1 is not required for the uptake of folates to function as vitamins.

### The loss of FOLR-1 causes changes in gene expression

Although the loss of FOLR-1 does not induce any detectable phenotypes, we asked how it affects gene expression. For this purpose, we performed RNA-seq analysis to compare gene expression in the L4 stage N2 and *folr-1(syb4116)* animals. Data analysis revealed that the expression of 451 and 447 genes are up- and downregulated, respectively, in *folr-1(syb4116)* animals (Fig. S5a, Supplementary Information file 2). Considering that FOLR-1::mNeonGreen is detectably expressed only in few cells (Fig. 1a and Fig. S1), the unexpectedly large number of differentially expressed genes presents three plausible explanations. First, FOLR-1 may be expressed in multiple tissues, but the sensitivity of the FOLR-1::mNeonGreen transgene may be insufficient for its detection. Second, FOLR-1 could exert non-cell autonomous effects on organismal physiology, and third, the observed changes in gene expression could be secondary effects. When performing KEGG pathway^37^ enrichment analysis for the genes upregulated in *folr-1(syb4116)* mutants (Supplementary Information file 2), we found that ribosome, the macromolecular machine mediating protein synthesis, shows the most significant enrichment (Fig. S5b). On the other hand, downregulated genes in *folr-1(syb4116)* mutants (Supplementary Information file 2) did not show any significantly enriched KEGG pathway. To validate the RNA-seq data, we performed qRT-PCR analysis of selected ribosomal genes that were found to be significantly upregulated in *folr-1(syb4116)* mutants in RNA-seq. Interestingly, when comparing with the RNA-seq data, qRT-PCR did not show as clear upregulation of ribosomal genes, as the expression of many genes was found to be unchanged in *folr-1* mutants (Fig. S6a and b). For further validation, we used a strain in which endogenous RPS-6, a ribosomal subunit that shows elevated expression in *folr-1* mutants (Fig. S6a, b and Supplementary Information file 2), is tagged with fluorescent wrmScarlet by CRISPR-Cas9-mediated genome editing. As expected, RPS-6 signal can be detected in every cell of L4 larvae, but we did not detect any differences between wild-type- and *folr-1(syb5135)* background (Fig. S6c). Similarly to RPS-6::wrmScarlet imaging, RPS-6 Western blots from whole-animal extracts of day 2 adults did not reveal any differences between N2 and *folr-1(syb4116)* mutants (Fig. S6d and e). Together, the data presented above provide strong evidence that, unlike the loss of folate transporter FOLT-1^23^, the depletion of *folr-1* does not affect health- or lifespan in *C. elegans*. On the other hand, given that the loss of FOLR-1 affects the expression of approximately 900 genes, this receptor likely plays a role in other physiological processes, as demonstrated, for example, in germ cell homeostasis^24^.

### *folr-1* overexpression is toxic for animals maintained on *E coli* HT115

As mentioned earlier, FR is overexpressed in multiple cancers^14,15,17–21^. For example, the expression of FOLR1 has been shown to be 100–300 times higher in breast, lung, kidney, and ovarian cancers when compared to healthy cells^18^. At the protein level, the highest FOLR1 expression has been detected in ovarian and brain carcinomas, in which FOLR1 level can be up to 30 and 14 times higher compared to normal cells, respectively^14^. Since FOLR1 overexpression in tumors has been associated with increased cancer progression and poor patient prognosis^17,38^, we asked how increased FR expression affects the physiology of *C. elegans*, in which all somatic cells in an adult animal are post-mitotic^39^. Since *lin-53* RNAi induces increased and ectopic FOLR-1 expression (Fig. 1b), and LIN-53 is required for normal lifespan^40^, we asked how *lin-53* knockdown affects the lifespan of *folr-1* mutants. Strikingly, whereas *lin-53* RNAi strongly shortens N2 lifespan, the *folr-1(syb5135)* mutation partially rescues this phenotype (Fig. 2g), suggesting that elevated FOLR-1 level explains, at least partly, the shortened lifespan upon reduced *lin-53* expression.

**Figure 3:**
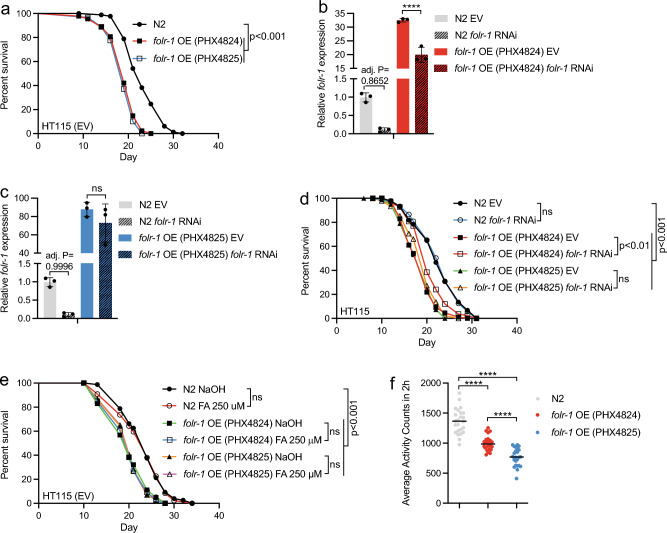
*folr-1* overexpression (OE) induces toxicity on *E. coli* HT115. (**a**) Lifespan of *folr-1* OE strains (PHX4824 and PHX4825) on HT115 (EV) compared to N2. Data are representative of at least three independent experiments. (**b**) *folr-1* qRT-PCR of *folr-1* overexpression (OE) strains PHX4824 and (**c**) PHX4825 upon control and *folr-1* RNAi. Data in (**b**) and (**c**) are from the same experiment divided into two graphs. Bars represent *folr-1* mRNA levels with error bars indicating mean ± SD of three biological replicates, each with three technical replicates (****p < 0.0001, one-way ANOVA with Tukey’s test). Notably, in (**b**) and (**c**), when comparing only N2 EV and N2 *folr-1* RNAi, Student’s t-test shows that *folr-1* RNAi significantly downregulates *folr-1* mRNA level (*p* < 0.001). The experiment was performed two times with similar results. (**d**) Lifespan of N2 and *folr-1* OE strains on *folr-1* RNAi. Data are representative of two independent experiments. (**e**) Lifespan of N2 and *folr-1* OE strains on plates supplemented with 250 μM folic acid (FA). The experiment was performed once. Statistical calculations for lifespan experiments were performed using the Cox-proportional hazard regression analysis. Lifespan statistics are reported in Supplementary Information file 1, Table S1. (**f**) Activity of day 4 adult N2 and *folr-1* OE strains measured with wMicroTracker. Each dot represents a group of 10 animals (n = 240 animals per condition). Data are combined from two independent experiments (****p < 0.0001, one-way ANOVA with Tukey’s test).

To study further whether the elevated FOLR-1 level decreases lifespan, we used two independent *folr-1* overexpression (OE) strains (PHX4824 and PHX4825). These strains carry extra copies of the *folr-1* gene under its own promoter sequence. To ensure efficient expression of the transgene, the *folr-1* 3′UTR was replaced with *unc-54* 3′UTR. Since *lin-53* RNAi experiments were performed on *E. coli* HT115 (Fig. 2g), the same bacterial strain was chosen for lifespan experiments with *folr-1* OE animals. Strikingly, we found that *folr-1* OE is toxic, as it decreases lifespan in both OE strains (Fig. 3a). To confirm that the toxicity is due to *folr-1* overexpression, we asked whether *folr-1* RNAi rescues the decreased lifespan of these strains. Before this experiment, we measured how efficiently *folr-1* RNAi downregulates *folr-1* mRNA level in *folr-1* OE strains. To ensure maximal knockdown efficiency, we placed L1 larvae of P0 generation on *folr-1* RNAi and extracted RNA from L4 larvae of the next generation (F1 generation), thereby exposing animals to *folr-1* RNAi for two generations. Interestingly, qRT-PCR analysis revealed that *folr-1* RNAi partially blunts *folr-1* overexpression in the *folr-1* OE strain PHX4824, but not in the *folr-1* OE strain PHX4825 (Fig. 3b and c). This may be because the *folr-1* OE strain PHX4825 shows higher *folr-1* expression than the PHX4824 strain when compared to N2 (Fig. 3b and c), and the high *folr-1* expression in the PHX4825 strain may saturate the organismal RNAi capacity to downregulate this mRNA. For the lifespan analysis, we performed two independent lifespan experiments with *folr-1* RNAi. In the first experiment, *folr-1* RNAi partially rescued the toxicity in the *folr-1* OE strain PHX4824 (Fig. 3d), while in the second experiment the decreased lifespan of the *folr-1* OE strain PHX4824 was fully rescued (see Supplementary Information file 1, Table S1). However, according to the qRT-PCR data showing that *folr-1* RNAi does not reduce *folr-1* mRNA level in the *folr-1* OE strain PHX4825 (Fig. 3c), *folr-1* RNAi does not rescue the toxicity of this strain (Fig. 3d and Supplementary Information file 1, Table S1). Together, these experiments strengthen the conclusion that increased *folr-1* expression is toxic for *C. elegans*.

Since high concentrations of folic acid have been shown to be toxic to *C. elegans* when maintained on OP50^33^, we asked whether *folr-1* OE induces toxicity due to excess absorption of folate. Therefore, we tested whether supplementing plates with folic acid leads to a further decrease in their lifespan. In our experiment, folic acid supplementation did not affect the lifespan of *folr-1* OE strains (Fig. 3e), suggesting that the negative effects of *folr-1* OE are not due to possible folate toxicity. However, since folic acid is degraded and affects *C. elegans* physiology indirectly via bacterial uptake of breakdown products^41^, we cannot rule out that *folr-1* OE toxicity is, at least partly, due to excess folate absorption. Finally, to test whether increased *folr-1* expression affects physical condition, we measured the activity of *folr-1* OE strains at day 4 of adulthood. In contrast to *folr-1* mutants (Fig. 2f), both strains show significant reduction in activity when compared to N2 (Fig. 3f). These data are a striking demonstration of how FR overexpression can deteriorate the animal’s health, thus providing possible explanation for why FR expression is tightly controlled in both *C. elegans* (Fig. 1a,b and Fig S1) and humans^13–15^.

### *folr-1* overexpression does not affect the lifespan of animals maintained on *E. coli* OP50

**Figure 4:**
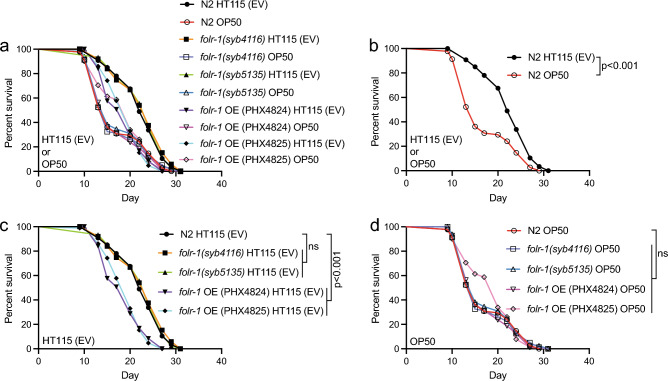
*folr-1* overexpression does not affect lifespan on *E. coli* OP50. (**a**) Lifespan of N2, *folr-1* OE strains (PHX4824 and PHX4825) and *folr-1* mutants (*folr-1(syb4116)* and *folr-1(syb5135)*) on HT115 (EV) and OP50. The data in (**a**) are divided into graphs that show (**b**) the lifespan of N2 on HT115 (EV) and OP50, and the lifespan of N2, *folr-1* OE strains (PHX4824 and PHX4825) and *folr-1* mutants (*folr-1(syb4116)* and *folr-1(syb5135)*) on (**c**) HT115 (EV) and (**d**) OP50. Data are representative of two independent experiments. Statistical calculations for lifespan experiments were performed using the Cox-proportional hazard regression analysis. Lifespan statistics are reported in Supplementary Information file 1, Table S1.

*C. elegans* is maintained on bacterial lawn, and the bacterial diet also forms its gut microbiota. Importantly, it has been shown that changes in microbial metabolism have a significant impact on longevity^5–7,42^. In this context, *E. coli* HT115 and OP50 are the two most used bacterial strains in *C. elegans* experiments, and even these bacteria, which differ in their metabolism and transcriptome, have different effects on lifespan^43,44^. Since *C. elegans* has a reduced lifespan when grown on OP50 compared to HT115 (on which the above-described lifespan experiments were performed)^5,43,44^, we asked how OP50 affects the longevity of *folr-1* mutants and OE strains. We found that N2, *folr-1(syb4116)* and *folr-1(syb5135)* mutants have a similar lifespan on OP50, which is shorter than on HT115 (Fig. 4a,b). Interestingly, although *folr-1* OE strains have a reduced lifespan on HT115 (Fig. 4a,c), their lifespan is similar to that of N2 and *folr-1* mutants on OP50 (Fig. 4a,d), demonstrating that *folr-1* OE induces toxicity in a diet-dependent manner.

**Figure 5:**
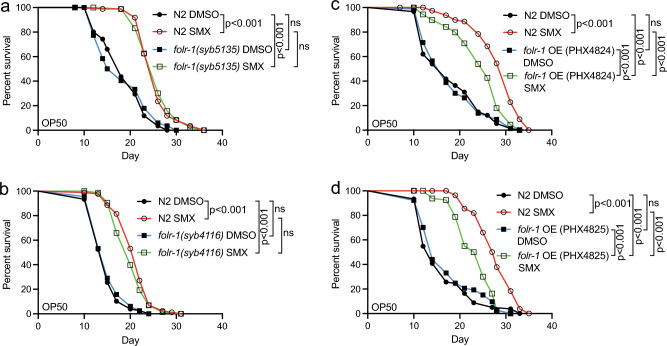
*folr-1* overexpression blunts the SMX-mediated longevity on OP50. (**a**) Lifespan of N2, *folr-1(syb5135)* and (**b**) *folr-1(syb4116)* on SMX-supplemented OP50 plates. Experiments in (**a**) and (**b**) were performed once. (**c**) Lifespan of N2, *folr-1* OE strain PHX4824 and (**d**) *folr-1* OE strain PHX4825 on SMX-supplemented OP50 plates. Data in both (**c**) and (**d**) are representative of two independent experiments. Statistical calculations for lifespan experiments were performed using the Cox-proportional hazard regression analysis. Lifespan statistics are reported in Supplementary Information file 1, Table S1.

To test whether the effect of *folr-1* OE on lifespan is affected by the metabolomic profile of diet/microbiota, we utilized sulfamethoxazole (SMX). SMX is a sulfonamide drug that extends *C. elegans* lifespan through the inhibition of microbial folate synthesis^6,7^. Importantly, the lifespan-extending effect of SMX is based on its ability to limit folate in *E. coli*, but not in *C. elegans*^7^. First, we found that the loss of FOLR-1 does not affect the SMX-induced lifespan extension (Fig. 5a and b). In contrast to *folr-1* mutants, we discovered that *folr-1* OE reduces the SMX-mediated longevity (Fig. 5c and d). These data suggest that *folr-1* OE induces toxicity through a mechanism that is, at least partially, dependent on bacterial folate synthesis within the diet/microbiota.

## Discussion

Our work show that, unlike the depletion of RFC homolog FOLT-1^23^, the loss of FOLR-1 does not affect health- or lifespan, thus supporting the statement by Chaudhari et al. that FOLR-1 is not essential for the uptake of folates to function as vitamins^24^. In contrast to *C. elegans*, FR ablation leads to embryonic lethality in mice^45^. Furthermore, it is well-established that FR dysfunction in humans, either through genetic mutation or autoantibodies blocking its function, causes birth defects and neurodevelopmental disorders in childhood^46–51^, thus demonstrating that the role of FR in development differs between species. Probably the main factor explaining this difference is that in mammals FOLR1 is required to transport folate via transcytosis across the blood-cerebrospinal fluid barrier^16^, whereas the ubiquitous expression of FOLT-1^22^ is sufficient to ensure developmental integrity in *C. elegans*. Nevertheless, the lack of phenotypes in adult *folr-1* mutants highlights FR as an attractive and safe target for interventions that inhibit its function.

Although the role of FR in human and *C. elegans* development differs, its restricted expression is a common feature in both species. Interestingly, it has been shown that histone variant H2A.Z.1 and histone chaperone ASF1a promote the expression of FOLR1 in cultured mouse neural precursor cells^52^. However, to our knowledge, it is not known which factors inhibit FR expression in a multicellular organism. Our data highlight the role of LIN-53/RbAp48 in restricting FOLR-1 expression, thus raising the question of whether this histone-binding protein regulates FR expression also in humans. Tightly controlled expression of FR in both human and *C. elegans* implies that this receptor is detrimental for organismal health when expressed at high levels. Indeed, we show here that ectopic FOLR-1 expression upon *lin-53* knockdown contributes to shortened lifespan (Figs. 1b and 2g), and that *folr-1* OE induces toxicity in a diet-dependent manner (Figs. 4, 5c and d).

Currently we cannot provide a mechanism for how elevated FOLR-1 level interacts with diet/microbiota to induce toxicity. As mentioned earlier, FOLR-1 mediates the biological effects of bacterial folates^24^, raising the possibility that *folr-1* OE increases the uptake of these folates from the co-cultured bacteria, which then at high doses have adverse effects on health. Since N2 animals have a shorter lifespan on OP50 compared to HT115 (Fig. 4a and b)^5,43,44^, it is possible that OP50 bacteria produce more toxic bacterial folates. Interestingly, metformin, a widely used drug to treat type 2 diabetes, promotes longevity on OP50 by disrupting bacterial folate and methionine cycles, whereas it does not extend lifespan on HT115^5^, thus supporting the hypothesis that OP50 contains an elevated level of toxic bacterial folates. On the other hand, since *folr-1* OE induces toxicity only on HT115 (Fig. 4a), an alternative hypothesis is that elevated FOLR-1 level may sensitize animals to HT115-produced bacterial folates, thus leading to decreased lifespan. In this context, Chaudhari et al. demonstrated that, in comparison to OP50, HT115 bacteria produce more stimulatory bacterial folates that promote FOLR-1-dependent germ cell proliferation^24^. Furthermore, our findings indicate that the lifespan extension observed with SMX-mediated inhibition of bacterial folate metabolism is less pronounced in *folr-1* OE animals compared to N2 (Fig. 5c and d), suggesting that the overexpressed FOLR-1 can uptake bacterial folates from the pool that is generated even in the presence of SMX, which then suppresses SMX-induced longevity. Importantly, although the hypothesis that *folr-1* OE causes toxicity due to increased uptake of toxic bacterial folates is intriguing, it should be noted that the validation of this hypothesis would require extensive testing of whether *folr-1* OE affects only SMX-mediated longevity and not, for example, the lifespan of long-lived mutants.

Regarding humans, the expression of FRs, and especially FOLR1, has been shown to be strongly increased in certain cancers^14,15,17–21^. Moreover, whereas RFC is considered as a putative tumor suppressor, FOLR1 is considered as a putative oncogenic factor in human malignancies^53^. Notably, aberrant gut microbiota contributes to the onset and progression of several maladies, including cancer^54–56^. Although we cannot draw direct conclusions from *C. elegans* experiments to human cancer patients, our data raise the possibility that an interaction between an aberrant gut microbiota and increased FR expression may contribute to the systemic deterioration of health in cancer patients. Finally, although we are left with many open questions, this work highlights the importance of restricted FOLR-1 expression for both physical condition and lifespan. Therefore, our data should encourage further studies on the role of this receptor in the organismal physiology, especially under conditions where it is overexpressed.

## Materials and methods

### *C. elegans* strains and maintenance

For all experiments *C. elegans* were maintained on NGM plates (peptone, P4963, Merck; agar, A4550, Merck; NaCl, 746398, Merck) seeded with *E. coli* HT115 bacteria carrying the empty vector (EV, a control vector for RNAi) or *E. coli* OP50. The N2 (Bristol) strain was used as the wild-type. N2, GMC101 and CL2122 strains were obtained from the Caenorhabditis Genetics Center (CGC). The *folr-1(syb4116)* X (PHX4116), *folr-1(syb5135)* X (PHX5135), *folr-1(syb4185)*[*folr-1::mNeonGreen*] X (PHX4185), *rps-6(syb7330[rps-6::wrmScarlet])* I (PHX7330) strains were created by using CRISPR/Cas9-mediated genome editing (SunyBiotech). All described crosses between genotypes were created within this study. The *folr-1* OE strains (PHX4824 and PHX4825, *Is[Pfolr-1::folr-1::unc-54 3’UTR, Pmyo-2::gfp]*) were created by using microinjection (SunyBiotech). Transgenes were integrated into the genome by gamma irradiation (SunyBiotech). The PHX4116 and PHX5135 strains were outcrossed two times with N2. The PHX4824 and PHX4825 were outcrossed six times with N2. Related sequences for strains created within this study can be found in Supplementary Information file 1.

### RNA interference (RNAi)

*Folt-1* RNAi was created by cloning a part of *folt-1* ORF from *C. elegans* cDNA by using the 5′-ataaccggtCCGTAAAGGAGTTTCGACCA-3′ and 5′-ataggtaccAAAATTGAAGCGACCAGTGC-3′ primers and ligating it into the L4440 vector. The *lin-53* and *folr-1* RNAi clones were taken from the Ahringer RNAi library. RNAi was performed by using the feeding protocol described earlier^57^. In *folr-1* RNAi experiments, animals were grown on RNAi for one generation (P0) before the experiment (qRT-PCR and lifespan analyses were performed with F1 generation).

### Imaging

For confocal imaging, *folr-1(syb4185)*[folr-1::mNeonGreen] X (PHX4185) animals were maintained at 20 °C and imaged as L4 larvae and as gravid day 1 adults. The animals were mounted on 2% agarose pads and immobilized by using levamisole hydrochloride (30 μM) diluted in M9 solution. A Leica SP8 upright confocal microscope with HC PL APO 63x/1.30 GLYC CORR CS2 objective was used for imaging. For the *lin-53* RNAi experiment, *folr-1(syb4185)*[folr-1::mNeonGreen] X (PHX4185) animals were grown on RNAi from hatching at 20 °C. L4 larvae animals were mounted on 2% agarose pads and immobilized by using levamisole hydrochloride (30 μM) diluted in M9 solution. Imaging was done with an Olympus BX63 microscope by using a 10 × objective. Similarly, *rps-6(syb7330[rps-6::wrmScarlet])* I (PHX7330) animals were mounted on 2% agarose pads as L4 larvae and immobilized by using levamisole hydrochloride (30 μM) diluted in M9 solution. Imaging was done with an Olympus BX63 microscope by using 10 × objective.

### Lifespan analyses

Except for one experiment done at 25 degrees Celsius (°C) (Fig. S4a), all *C. elegans* lifespan experiments were done at 20 °C. Lifespan experiments were initiated by letting gravid hermaphrodites (P0 generation) to lay eggs on NGM agar plates, and the F1 generation was scored for lifespan. Alternatively, animals were bleached and allowed to hatch overnight in M9 before plating L1 larvae on experimental plates. These two alternative ways to initiate lifespan did not affect the conclusions made from the experiments. Lifespan experiments were performed on either *E. coli* HT115 (carrying the empty vector, EV) or *E. coli* OP50. For folate supplementation experiments, folic acid (FA, Merck, #F8758) and 5-methyltetrahydrofolate (5-MTHF, Merck, #M0132) were added to NMG media at indicated concentrations. Sulfamethoxazole (SMX, Merck, #S7507) was added to NMG media at a final concentration of 128 μg/ml^6,7^. At the L4 larval stage animals were transferred to plates containing 5-Fluorouracil (10 µM) (Merck, #F6627) to prevent progeny production. Animals that had exploded vulva or that crawled off the plate were censored. Animals were counted as dead if there was no movement after poking with a platinum wire. Lifespans were checked every 1–3 days. Mean lifespan ± standard error (SE) is reported in Supplementary Information file 1, Table S1.

### Activity measurement

Animals were synchronized by bleaching and plated as L1 larvae on NGM agar plates seeded with *E. coli* HT115 (EV), which were kept at 20 °C. At L4 larval stage animals were transferred to plates containing 5-Fluorouracil (10 µM) (Merck, #F6627) to prevent progeny production. The activity of N2, *folr-1* mutants and *folr-1* OE strains was measured on day 4 of adulthood. GMC101 and CL2122 (and crosses of these strains with *folr-1(sy4116)* mutant) were transferred to 25 °C on day 1 of adulthood, and the activity was measured on day 2 of adulthood. For activity measurement, 10 animals were placed in a single well of a 96-well plate containing 100 μl of M9 solution. 10–12 wells were used per experiment for each strain. Activity was measured for two hours with wMicroTracker (InVivo Biosystems).

### Progeny count

Single L4 stage N2 and *folr-1(syb4116)* mutants were placed on 10 small agar plates. Animals were transferred to new plates daily during the reproduction period, and viable offspring were counted.

### RNA-seq

N2 and *folr-1(syb4116)* animals were synchronized by bleaching and plated as L1 larvae on NGM agar plates seeded with *E. coli* HT115 (EV). Animals were collected at the L4 larval stage (three biological replicates for both strains) and frozen in liquid nitrogen. Total RNA was extracted with TRIzol Reagent (ThermoFisher Scientific, #15596018). Samples were sent to Novogene for library construction, quality control and sequencing. In short, mRNA was purified from total RNA using poly-T oligo-attached magnetic beads. After the fragmentation, the first strand cDNA was synthesized using random hexamer primers, followed by the second strand cDNA synthesis using either dUTP for directional library or dTTP for non-directional library. The library preparations were sequenced on an Illumina platform and paired-end reads were generated. To obtain clean reads, raw data (raw reads) of FASTQ format were firstly processed through fastp. Paired-end clean reads were mapped to the reference genome using HISAT2 software. FeatureCounts was used to count the read numbers mapped of each gene. Consequently, RPKM of each gene was calculated based on the length of the gene and reads count mapped to this gene. Prior to differential gene expression analysis, for each sequenced library, the read counts were adjusted by Trimmed Mean of M- values (TMM) through one scaling normalized factor. Differential expression analysis between two conditions (three biological replicates per condition) was performed using DESeq2 R package^58^. The resulting P values were adjusted using the Benjamini and Hochberg’s approach for controlling the False Discovery Rate (FDR). Genes with an adjusted *P* value < 0.05 found by DESeq2 were assigned as differentially expressed. Differential expression analysis of two conditions was performed using the edgeR R package^59^. The P values were adjusted using the Benjamini and Hochberg methods. A corrected pvalue of 0.005 and |log2^(Fold Change)^| of 1 were set as the threshold for significantly differential expression. Differentially expressed genes can be found from Supplementary Information file 2. KEGG pathway enrichment analysis was done with the clusterProfiler R package. The RNA-seq data are available in the Gene Expression Omnibus (GEO) database repository (GSE227272).

### Quantitative RT-PCR (qRT-PCR)

Animals were synchronized by bleaching and plated as L1 larvae on NGM agar plates seeded with *E. coli* HT115 (EV), which were kept at 20 °C. Animals were collected at the L4 stage and frozen in liquid nitrogen. TRIzol Reagent (ThermoFisher Scientific, #15596018) was used to extract RNA. cDNA synthesis was done with the QuantiTect Reverse Transcription Kit (Qiagen, #205313) and qRT-PCR reactions were run with HOT FIREPol SolisGreen qPCR Mix-reagent (Solis BioDyne, #08-46-00001) using the CFX384 machine (Bio-Rad). qRT-PCR data were normalized to the expression of *cdc-42* and *pmp-3*. qRT-PCR oligos used in this study are provided in Supplementary Information file 1, Table S2. qRT-PCR experiments were performed with three biological replicates, with three technical replicates for each biological replicate. Analysis of ribosomal subunit expression was performed once with each *folr-1* mutant, and the analysis of *folr-1* RNAi efficiency in N2 and *folr-1* OE strains was performed twice with similar results. Statistical significances were analyzed by using Student’s t-test and one-way ANOVA.

### Western blot

Animals were synchronized by bleaching and plated as L1 larvae on NGM agar plates seeded with *E. coli* HT115 (EV), which were kept at 20 °C. At the L4 larval stage, animals were transferred to plates containing 5-Fluorouracil (10 µM) (Merck, #F6627) to prevent progeny production. N2, and *folr-1(syb4116)* mutants were collected on day 2 of adulthood and frozen in liquid nitrogen. GMC101 and CL2122 (and crosses of these strains with *folr-1(sy4116)* mutant) were transferred to 25 °C on day 1 of adulthood, collected on day 2 of adulthood, and frozen in liquid nitrogen. Animals were lysed in a protease inhibitor cocktail (ThermoFisher Scientific, #78430)-supplemented urea solution (Merck, #51457) by grinding with a plastic pestle in 1.5 ml Eppendorf tubes. Lysates were resolved on 4–15% precast polyacrylamide gels (Bio-Rad, #4561083). Immun-Blot PVDF Membrane (Bio-Rad, #1620177) was used for blotting. Purified anti-β-Amyloid, 1–16 Antibody (6E10) (used with a 1:1000 dilution) was purchased from BioLegend. Anti-S6 Ribosomal Protein Antibody (used with a 1:1000 dilution) was purchased from Merck (#ZRB1172). α-tubulin antibody (used with a 1:5000 dilution) was purchased from Merck (#T5168). Clarity Western ECL Substrate (Bio-Rad, #1705061) and ChemiDoc MP-imager (Bio-Rad) were used for protein detection in Western blot.

### Statistical analysis

Statistical analyses for qRT-PCR data were carried out in GraphPad Prism and Excel, and the data represent the mean of three biological replicates ± standard deviation (SD). Statistical analyses for activity measurements and Western blot data were carried out in GraphPad Prism and Excel, respectively. Statistical details can be found in the figures and figure legends. Statistical calculations for lifespan experiments were carried out in R by using the Cox-proportional hazard regression analysis. Statistical details for the lifespan data can be found in Supplementary Information file 1, Table S1.

### Supplementary Information

Supplementary Information 1.

Supplementary Figure S2.

Supplementary Figure S3.

Supplementary Information 2.

## Data Availability

The RNA-seq data generated during the current study are available in the Gene Expression Omnibus (GEO) database repository (GSE227272). *C. elegans* strains generated during the current study are available upon request (olli.matilainen@helsinki.fi).
