# Supplementary material for: Folate receptor overexpression induces toxicity in a diet-dependent manner in C. elegans
Source: Sci Rep. 2024 Jan 11;14:1066. doi: 10.1038/s41598-024-51700-9 (PMC10784478; doi:10.1038/s41598-024-51700-9)
Supplement: Supplementary file 1 — Supplementary Information 1. [file 41598_2024_51700_MOESM1_ESM.pdf]

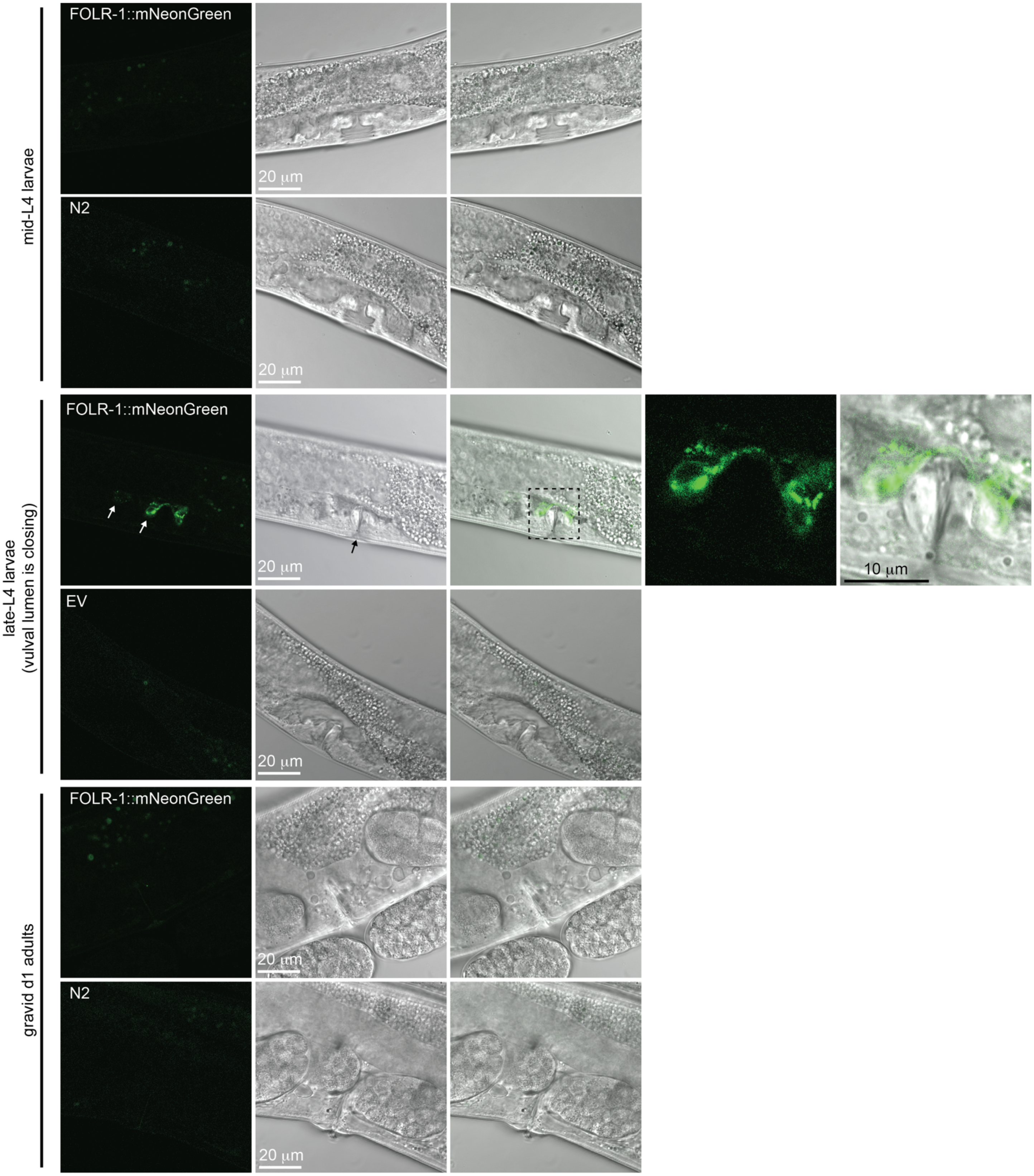

Figure S1. Confocal images of mid-L4 and late L4 larvae and gravid day 1 adult expressing FOLR-1::mNeonGreen. N2 is used as a control to recognize background signal. Late L4 larvae (middle panels) are imaged at the point when FOLR-1::mNeonGreen signal is strong (vulval lumen is closing, which is a later L4

sub-stage compared to the L4 larvae shown in Fig. 1a.) White arrows indicate FOLR-1::mNeonGreen localization and the black arrow marks the closing vulval lumen. The dashed square marks the magnified area shown on the right. All FOLR-1::mNeonGreen confocal images shown in this manuscript were taken with the same settings during the same imaging session, facilitating the comparison of the images.

Figure S2 (figure as separate file). *folr-1* expression by cell type. Figure has been downloaded from WormSeq.

Figure S3 (figure as separate file). *folr-1* expression dot plot. Figure has been downloaded from WormSeq.

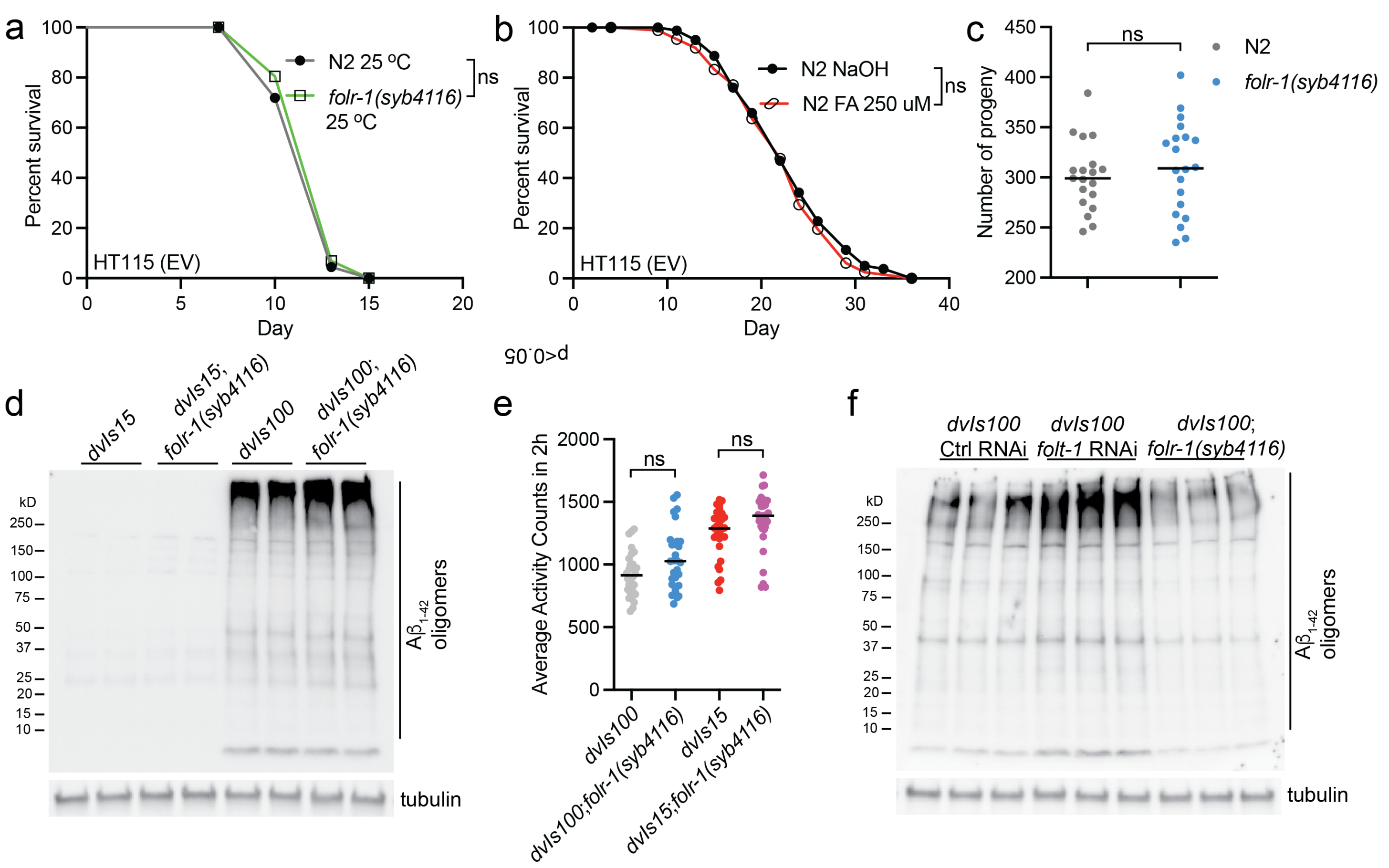

Figure S4. (**a**) Lifespan of N2 and *folr-1(syb4116)* mutants at 25 degrees Celsius (25 ºC). The experiment was performed once. (**b**) Lifespan of N2 animals on plates supplemented with 250 mM folic acid (FA). Data are representative of two independent experiments. Statistical calculations for lifespan experiments were performed using the Cox-proportional hazard regression analysis. Lifespan statistics are reported in Supplementary Information file 1, S1 Table. (**c**) Brood size of N2 and *folr-1(syb4116)* mutants. Data are combined from two

independent experiments. Statistical significance was calculated with an unpaired Student's *t*-test. (**d**) Amyloid beta (Aβ) Western blot and (**e**) activity of day 2 adult Aβ-expressing strain (GMC101, expresses transgene *dvIs100*) and control strain (CL2122, expresses transgene *dvIs15*) in wild-type- and *folr-1(syb4116)* background measured with wMicroTracker. In (**e**), each dot represents a group of 10 animals (n = 300 animals per condition). Data are combined from three independent experiments (**p < 0.01, ****p < 0.0001, one-way ANOVA with Tukey's test). (**f**) Aβ Western blot of Aβ-expressing strain upon control- and *folt-1* RNAi, and with *folr-1(syb4116)* background. Western blots in (**d**) and (**f**) were repeated at least twice with similar results.

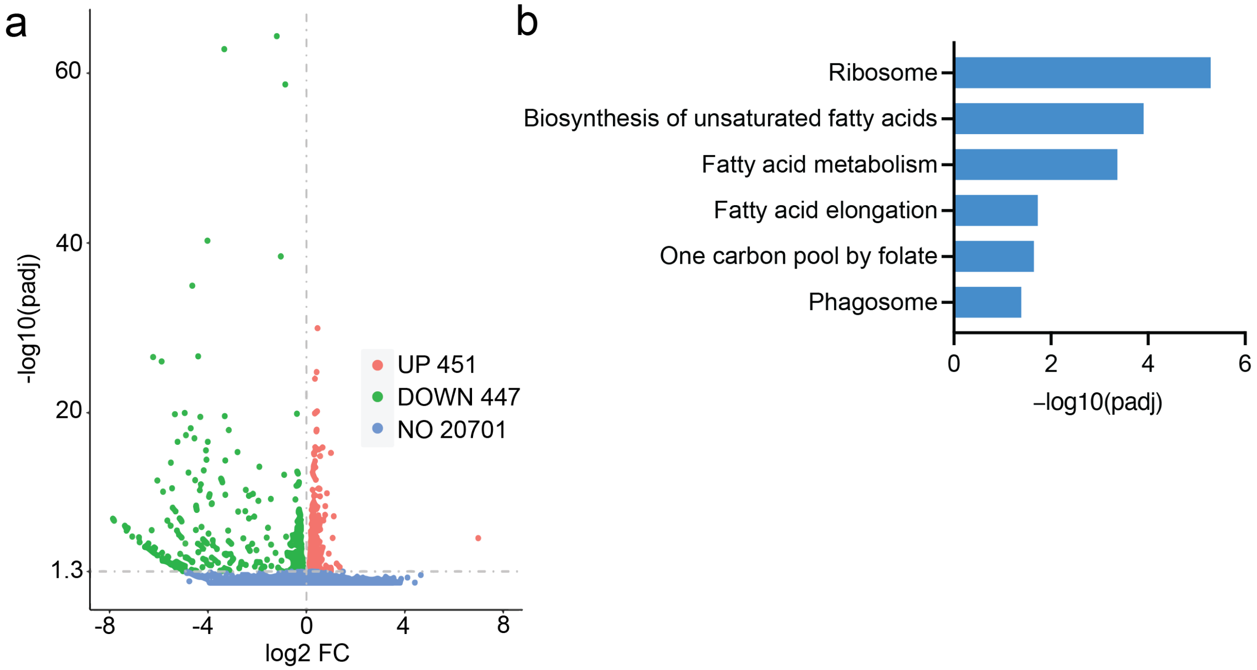

Figure S5. (**a**) Volcano plot showing differentially expressed genes in L4 stage *folr-1(syb4116)* mutants compared to N2. (**b**) Enriched KEGG pathways among upregulated genes in *folr-1(syb4116)* mutants. Lists of differentially expressed genes are shown in Supplementary Information file 2.

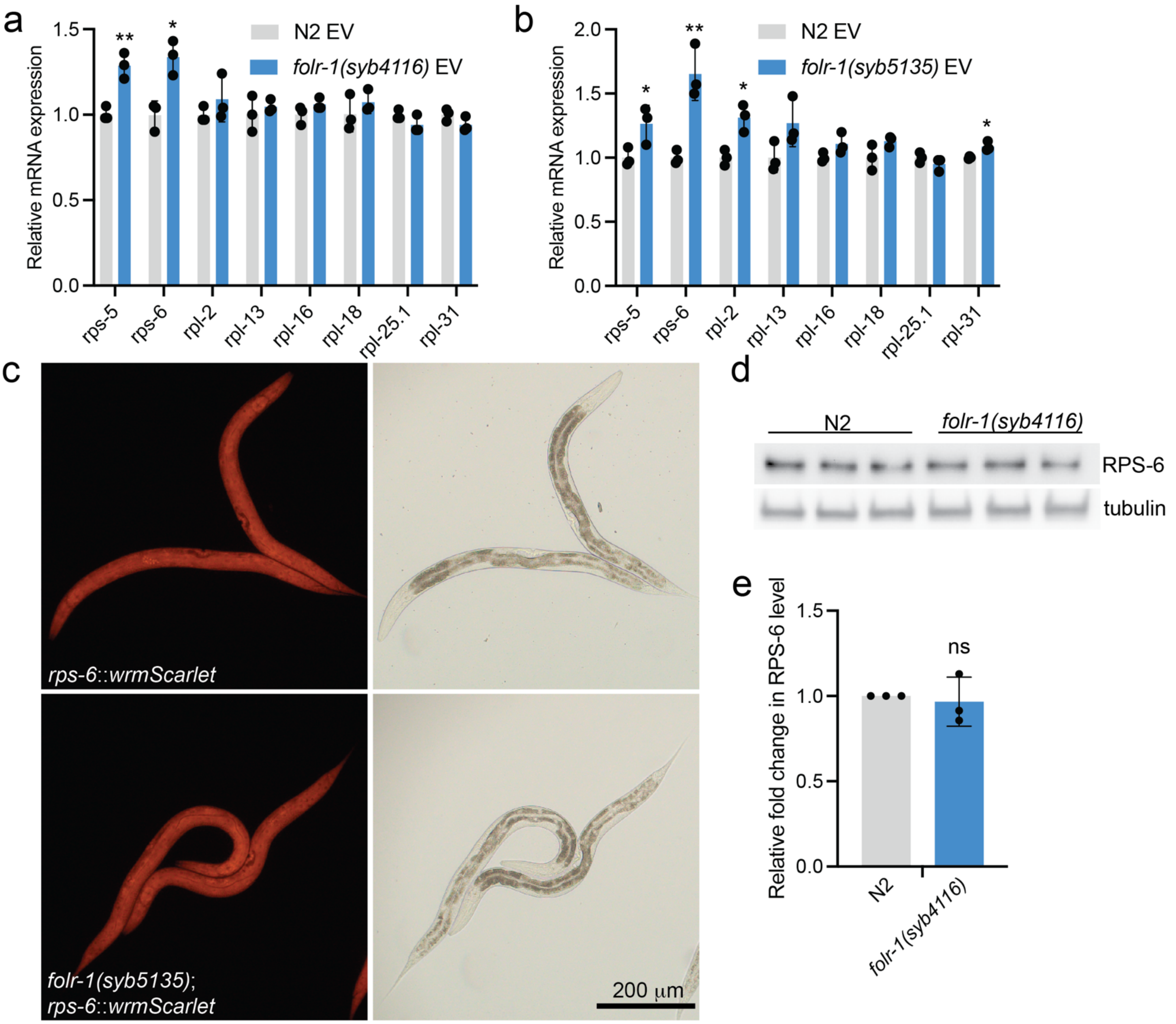

Figure S6. (**a**) qRT-PCR of selected ribosome subunits in L4 stage *folr-1(syb4116)* and (**b**) *folr-1(syb5135)* mutants compared to N2. Bars represent mRNA levels relative to N2 with error bars indicating mean ± SD of three biological replicates, each with three technical replicates (*p < 0.05, **p < 0.01, unpaired Student's t-test). Experiments in (**a**) and (**b**) were performed once. (**c**) Representative images of late L4 stage animals expressing RPS-6::wrmScarlet-fusion protein in wild-type- and *folr-1(syb5135)* background. (**d**) Representative Western blot of ribosomal RPS-6 in day 2 adult N2 and *folr-1(syb4116)* mutants. The blot shows three technical replicates from one biological replicate. (**e**) Quantification of RPS-6 Western blots of day 2 adult N2 and *folr-1(syb5135)* mutants. Bars represent the fold change of tubulin-normalized RPS-6 level relative to N2 with error bars

indicating mean ± SD of three biological replicates (statistical significance calculated with unpaired Student's t-test).

Table S1. Individual replicates of *C. elegans* lifespan experiments.

Genotype, RNAi and treatment	mean lifespan ± SE (days)	variation compared to control (%)	*P*-values against control	N
**Figure 2b**				
N2 (HT115, EV) (data used also in Figs. 2B and 2D)	20.6 ± 0.53			87
folr-1(syb5135) (HT115, EV) (data used also in Figs. 2B and 2D)	20.9 ± 0.53	+ 1.4	0.64590	84
N2 (HT115, EV) (data used also in Fig. 2E)	21 ± 0.55			88
folr-1(syb5135) (HT115, EV) (data used also in Fig. 2E)	21.4 ± 0.53	+ 1.9	0.67394	84
**Figure 2c**				
N2 (HT115, EV)	19.7 ± 0.51			80
folr-1(syb4116) (HT115, EV)	20.6 ± 0.37	+ 4.4	0.853	84
N2 (HT115, EV)	22.1 ± 0.55			80
folr-1(syb4116) (HT115, EV)	21.8 ± 0.57	+ 4.4	0.838	82
**Figure 2d**				
N2 (HT115, EV) (data used also in Figs. 2B and 2E)	20.6 ± 0.53			87
N2 (HT115, EV) FA 10 uM	21.4 ± 0.55	+ 3.7	0.53591	85
folr-1(syb5135) (HT115, EV)	20.9 ± 0.53	+ 1.4	0.64590	84

(data used also in Figs. 2B 2E)				
folr-1(syb5135) (HT115, EV) FA 10 uM	21.7 ± 0.54	+ 5<br># + 3.7	0.11552<br># 0.291	86
N2 (HT115, EV)	19.5 ± 0.44			91
N2 (HT115, EV) FA 10 uM	19.2 ± 0.48	- 1.5	0.793	81
folr-1(syb4116) (HT115, EV)	20.6 ± 0.44	+ 5.3	0.202	80
folr-1(syb4116) (HT115, EV) FA 10 uM	18.9 ± 0.62	- 3.1<br># - 8.3	0.495<br># 0.565	76

Figure 2e

N2 (HT115, EV)	20.5 ± 0.55			63
N2 (HT115, EV) 5-MTHF 100 nM	21.9 ± 0.59	+ 6.4	0.0451	70
N2 (HT115, EV)	20.9 ± 0.49			80
N2 (HT115, EV) 5-MTHF 100 nM	23.1 ± 0.44	+ 9.5	0.00253	85
N2 (HT115, EV)<br>(data used also in Figs. 2B and 2D)	20.6 ± 0.53			87
N2 (HT115, EV) 5-MTHF 100 nM	22.2 ± 0.62	+ 7.2	0.00587	85
folr-1(syb5135) (HT115, EV)<br>(data used also in Figs. 2B and 2D)	20.9 ± 0.53	+ 1.4	0.64590	84
folr-1(syb5135) (HT115, EV) 5-MTHF 100 nM	22.9 ± 0.58	+ 10<br># + 8.7	0.00194<br># 0.0126	82
N2 (HT115, EV)<br>(data used also in Fig. 2B)	21 ± 0.55			88
folr-1(syb5135) (HT115, EV)<br>(data used also in Fig. 2B)	21.4 ± 0.53	+ 1.9	0.67394	84
folr-1(syb5135) (HT115, EV) 5-MTHF 100 nM	23.5 ± 0.58	+ 10.6<br># + 8.9	0.00105<br># 0.00194	84

Figure 2g

N2 (HT115) EV	23.2 ± 0.56			80
N2 (HT115) *lin-53* RNAi	19.2 ± 0.35	- 17.2	3.97e-16	88
folr-1(syb5135) (HT115) EV	22.7 ± 0.53	- 2.2	0.49370	85
folr-1(syb5135) (HT115) *lin-53* RNAi	22.1 ± 0.4	- 4.7<br># - 2.6<br>° + 13.1	0.00237<br># 0.0207<br>° 3.52e-09	80
N2 (HT115) EV	23 ± 0.53			83
N2 (HT115) *lin-53* RNAi	19.1 ± 0.35	- 17	7.91e-13	85
folr-1(syb5135) (HT115) EV	22.4 ± 0.56	- 2.6	0.46850	81
folr-1(syb5135) (HT115) *lin-53* RNAi	21.6 ± 0.45	- 6.1<br># - 3.6<br>° + 11.6	0.00328<br># 0.0304<br>° 4.99e-06	79
**Figure 3a**				
N2 (HT115, EV)	19.5 ± 0.51			88
folr-1 OE (PHX4824) (HT115, EV)	17.1 ± 0.33	- 12.3	1.19e-06	88
folr-1 OE (PHX4825) (HT115, EV)	15.7 ± 0.31	- 19.5	2.82e-12	90
N2 (HT115, EV)	21.5 ± 0.59			85
folr-1 OE (PHX4824) (HT115, EV)	19.3 ± 0.36	- 10.2	0.000316	69
N2 (HT115, EV)	23.2 ± 0.57			83
folr-1 OE (PHX4825) (HT115, EV)	20.1 ± 0.54	- 13.4	3.63e-06	74
N2 (HT115, EV)	20.8 ± 0.47			87
folr-1 OE (PHX4825) (HT115, EV)	16.3 ± 0.33	- 21.6	6.55e-14	89
N2 (HT115, EV)	22.7 ± 0.47			86
folr-1 OE (PHX4824) (HT115, EV)	19.7 ± 0.4	- 13.2	1.28e-08	86
folr-1 OE (PHX4825) (HT115, EV)	19.5 ± 0.44	- 14.1	5.92e-08	77

N2 (HT115, EV)	23.2 ± 0.41			82
folr-1 OE (PHX4824) (HT115, EV)	19.2 ± 0.34	- 17.2	2.87e-12	88
folr-1 OE (PHX4825) (HT115, EV)	19 ± 0.3	- 18.1	1.70e-14	89
**Figure 3d**				
N2 (HT115) EV	22.5 ± 0.52			88
N2 (HT115) *folr-1* RNAi	22.7 ± 0.5	+ 0.9	0.991422	88
fol)r-1 OE (PHX4824) (HT115) EV	18.6 ± 0.38	- 17.3	1.97e-11	87
folr-1 OE (PHX4824) (HT115) *folr-1* RNAi	20.7 ± 0.42	- 8<br>& + 10.1	0.000931<br>& 0.00109	83
folr-1 OE (PHX4825) (HT115) EV	18.3 ± 0.38	- 18.7	6.18e-13	81
folr-1 OE (PHX4825) (HT115) *folr-1* RNAi	19.1 ± 0.39	- 15.1<br>@ + 4.2	1.16e-09<br>@ 0.131	88
N2 (HT115) EV	20.7 ± 0.54			85
N2 (HT115) *folr-1* RNAi	20.7 ± 0.51	+ 0	0.704999	82
folr-1 OE (PHX4824) (HT115) EV	18.4 ± 0.44	- 11.1	0.000127	86
folr-1 OE (PHX4824) (HT115) *folr-1* RNAi	20.2 ± 0.5	- 2.4<br>& + 8.9	0.312942<br>& 0.00542	81
folr-1 OE (PHX4825) (HT115) EV	17.8 ± 0.37	- 14	6.44e-08	81
folr-1 OE (PHX4825) (HT115) *folr-1* RNAi	18.7 ± 0.32	- 9.7<br>@ + 4.8	9.57e-06<br>@ 0.25	85
**Figure 3e**				
N2 (HT115, EV) NaOH	23.6 ± 0.48			80
N2 (HT115, EV) FA 250 uM	22.9 ± 0.54	- 3	0.788	85
folr-1 OE (PHX4824) (HT115, EV) NaOH	20 ± 0.46	- 15.3	3.74e-07	84
folr-1 OE (PHX4824) (HT115, EV) FA 250 uM	19.9 ± 0.42	- 15.7<br>& - 0.5	3.72e-08<br>& 0.663	86

folr-1 OE (PHX4825) (HT115, EV) NaOH	20 ± 0.42	- 15.3	9.57e-08	81
folr-1 OE (PHX4825) (HT115, EV) FA 250 uM	20.1 ± 0.44	- 14.8<br>@ - 0.5	5.36e-07<br>@ 0.848	74
**Figure 4a-4d**				
N2 HT115 (EV)	24 ± 0.58			85
N2 OP50	19.2 ± 0.76	- 20	0.000311	82
folr-1(syb5135) HT115 (EV)	24.1 ± 0.61	+ 0.4<br>¶ + 20.3	0.589306<br>¶ 5.33e-05	87
folr-1(syb5135) OP50	19.7 ± 0.72	- 17.9<br>¶ + 2.5	0.000899<br>¶ 0.7627	83
fol)r-1 OE (PHX4824) HT115 (EV)	20.5 ± 0.57	- 14.6<br>¶ + 6.3	0.000644<br>¶ 0.7717	88
folr-1 OE (PHX4824) OP50	17.6 ± 0.68	- 26.7<br>¶ - 8.3	3.76e-08<br>¶ 0.0597	77
folr-1 OE (PHX4825) HT115 (EV)	19.9 ± 0.48	- 17.1<br>¶ + 3.5	3.63e-06<br>¶ 0.3898	88
folr-1 OE (PHX4825) OP50	19.3 ± 0.67	- 19.6<br>¶ + 0.5	3.88e-05<br>¶ 0.6615	84
N2 HT115 (EV)	22.5 ± 0.54			86
N2 OP50	17 ± 0.66	- 24.4	1.21e-06	76
folr-1(syb4116) HT115 (EV)	22.7 ± 0.57	+ 0.9<br>¶ + 25.1	0.512<br>¶ 7.79e-08	86
folr-1(syb4116) OP50	16.8 ± 0.64	- 25.3<br>¶ - 1.2	2.40e-06<br>¶ 0.853	80
folr-1(syb5135) HT115 (EV)	22.7 ± 0.53	+ 0.9<br>¶ + 25.1	0.843<br>¶ 5.93e-07	87
folr-1(syb5135) OP50	17.4 ± 0.65	- 22.7<br>¶ + 2.3	2.26e-05<br>¶ 0.511	81
fol)r-1 OE (PHX4824) HT115 (EV)	18.5 ± 0.47	- 17.8	3.78e-06	86

		¶ + 8.1	¶ 0.676	
folr-1 OE (PHX4824) OP50	16.8 ± 0.6	- 25.3<br>¶ - 1.2	1.54e-07<br>¶ 0.759	80
folr-1 OE (PHX4825) HT115 (EV)	19 ± 0.43	- 15.6<br>¶ + 10.5	8.01e-06<br>¶ 0.579	85
folr-1 OE (PHX4825) OP50	18.6 ± 0.6	- 17.3<br>¶ + 8.6	5.69e-05<br>¶ 0.402	77
**Figure 5a**				
N2 (OP50) DMSO	18.7 ± 0.52			85
N2 (OP50) SMX (128 μg/ml)	25.7 ± 0.39	+ 27.2	2.65e-15	85
folr-1(syb5135) (OP50) DMSO	18.2 ± 0.61	- 2.7<br>* - 29.2	0.275<br>* 1.72e-12	84
folr-1(syb5135) (OP50) SMX (128 μg/ml)	25.7 ± 0.52	+ 27.2<br># + 29.2<br>* 0	1.01e-15<br># 2.16e-11<br>* 0.841	83
**Figure 5b**				
N2 (OP50) DMSO	14.8 ± 0.32			79
N2 (OP50) SMX (128 μg/ml)	21 ± 0.38	+ 29.5	<2e-16	85
folr-1(syb4116) (OP50) DMSO	15.2 ± 0.34	+ 2.6<br>* - 27.6	0.399<br>* <2e-16	83
folr-1(syb4116) (OP50) SMX (128 μg/ml)	20.2 ± 0.41	+ 26.7<br># + 24.8<br>* - 3.8	<2e-16<br># 2.54e-13<br>* 0.286	73
**Figure 5c**				
N2 (OP50) DMSO	18 ± 0.72			78
N2 (OP50) SMX (128 μg/ml)	28.5 ± 0.57	+ 36.8	< 2e-16	79
fol)r-1 OE (PHX4824) (OP50) DMSO	18.1 ± 0.69	+ 0.6<br>* + 36.5	0.64<br>* < 2e-16	81

fol)r-1 OE (PHX4824) (OP50) SMX (128 μg/ml)	24.6 ± 0.6	+ 26.2<br>& + 26.4<br>* - 13.7	1.56e-07<br>& 1.03e-06<br>* 3.86e-06	89
**Figure 5d**				
N2 (OP50) DMSO	16.1 ± 0.63			85
N2 (OP50) SMX (128 μg/ml)	27.7 ± 0.46	+ 41.9	< 2e-16	82
fol)r-1 OE (PHX4825) (OP50) DMSO	17 ± 0.69	+ 5.3<br>* + 38.6	0.589<br>* < 2e-16	74
fol)r-1 OE (PHX4825) (OP50) SMX (128 μg/ml)	23.2 ± 0.45	+ 30.6<br>@ + 26.7<br>* - 16.2	1.45e-06<br>@ 3.03e-05<br>* 1.34e-08	80
**Figure 5c-5d**				
N2 (OP50) DMSO	18.9 ± 0.78			81
N2 (OP50) SMX (128 μg/ml)	28.2 ± 0.63	+ 33	< 2e-16	80
fol)r-1 OE (PHX4824) (OP50) DMSO	18.3 ± 0.69	- 3.2<br>* - 35.1	0.131385<br>* < 2e-16	77
fol)r-1 OE (PHX4824) (OP50) SMX (128 μg/ml)	23.9 ± 0.67	+ 20.9<br>& + 23.4<br>* - 15.2	0.000195<br>& 1.08e-07<br>* 7.50e-07	77
fol)r-1 OE (PHX4825) (OP50) DMSO	19.2 ± 0.65	+ 1.6<br>* - 31.9	0.335175<br>* < 2e-16	78
fol)r-1 OE (PHX4825) (OP50) SMX (128 μg/ml)	24.2 ± 0.56	+ 21.9<br>@ + 20.7<br>* - 14.2	0.001212<br>@ 4.08e-06<br>* 4.66e-08	83
**Figure S2a**				
N2 (HT115, EV) 25 ºC	12.2 ± 0.16			89
folr-1(syb4116) (HT115, EV) 25 ºC	12.6 ± 0.15	+ 3.2	0.332	87
**Figure S2b**				

N2 (HT115, EV) NaOH	19 ± 0.33			87
N2 (HT115, EV) FA 250 uM	18.8 ± 0.37	- 1.1	0.977	90
N2 (HT115, EV) NaOH	22.9 ± 0.64			79
N2 (HT115, EV) FA 250 uM	22.2 ± 0.63	- 3.1	0.429	82

# Compared to untreated *folr-1* mutant

° Compared to *lin-53* RNAi-treated N2

& Compared to control-treated *folr-1* OE (PHX4824) strain

@ Compared to control-treated *folr-1* OE (PHX4825) strain

¶ Compared to OP50-treated N2

* Compared to SMX-treated N2

Table S2. Oligonucleotide sequences used in qRT-PCR

Gene	Forward (5' → 3')	Reverse (5' → 3')
cdc-42	CTGCTGGACAGGAAGATTACG	CTCGGACATTCTCGAATGAAG
pmp-3	GTTCCCGTGTTCATCACTCAT	ACACCGTCGAGAAGCTGTAGA
rps-5	GAGAAGTCCGCCAAGTACCT	TGATCTCAAAGGCGTGCTTG
rps-6	TGAGACTTAACTTCGCCTACCC	CACTCGTCACCGAGAGCAT
rpl-2	AGGAGCTGGTGGAATCTTCA	GGTCGTGGATGATGTCCTTC
rpl-13	CCACGTGGAAACCAAATGTT	GCCTTAGCTTGACGGTTCTG
rpl-16	TGACGGAAAGAACCACCTTC	GGGCTGGGTTGATGTTACAA
rpl-18	ACGCACTGGAGAGAAGTTCA	TCGAGAGGGTGACAACAGTC
rpl-25.1	CATCATCCAACACCCATTGA	GAGGGCTTGGACGTTGTAGA
rpl-31	AAGAAGTTCGCCAAGATCCA	GGAGAGACGGACACGAACTC
folr-1	GAGTTTAACAAGAACGTAGAG	TTTAGGCTGTTGATTATCAGTG

Sequences of deletions and insertions used in this study

Strain name and genotype: PHX4116 *folr-1(syb4116)*

-:deletion site
folr-1 exons are labeled with yellow and orange.

> *syb4116* (-4bp)

aggtactagcctactgtgagcccatataattccaacagtatattttccagttgaaattctaagcgctttccaagggtgaaatagtatttttaaattgtcgtttgtgagtcatcataaatatata
tgaaaatttgcttaggagagatgaaggtcacgcccatttgttatgagtcacaatcaaccgtactcgattttgtgcccctcttcttctctatttaaatgggatatctttgtttataacaatcattt
tttaattccttgctgttctcaaacaaaattgccgataattatttttcagaaATGAGATTCAAACTTGTTTGTCTTTTTTTAAGTCTATTTGCTCTT
CCGAGTGTGTCCCTC**TAG**ACATGAGCAAGTGGATGCCAGACACCCGGACTCACTCGATGGTGAAGCGGCAA
TGCGCAATGTGCATTGCCGGCGATTTTgtgagtttttttttttggctgaaagttttaatcaattatttattgtcacagTTGGACAAAAAGGATCT
GATGGTGGATGTATCTATTGGCCAGATGATTGTGTGGAACGATGAATGGGATTTAACAGACTGTTGGACTG
GGGATGTCGTCATCAAAGAATGTGCCTCGGCATGTGTCTCCATATACACCAAGTCGAAAACCCGTGAAGGA
TGGTTTTGGACAAgtgggctcaattgatcgtattttaactttttttcaaaaaattttcagGTGTCCTTATGGATTGTTCCGAGGC

> wild type

aggtactagcctactgtgagcccatataattccaacagtatattttccagttgaaattctaagcgctttccaagggtgaaatagtatttttaaattgtcgtttgtgagtcatcataaatatata
tgaaaatttgcttaggagagatgaaggtcacgcccatttgttatgagtcacaatcaaccgtactcgattttgtgcccctcttcttctctatttaaatgggatatctttgtttataacaatcattt
tttaattccttgctgttctcaaacaaaattgccgataattatttttcagaaATGAGATTCAAACTTGTTTGTCTTTTTTTAAGTCTATTTGCTCTT
CCGAGTGTGTCCCTCACAT**GGG**ACATGAGCAAGTGGATGCCAGACACCCGGACTCACTCGATGGTGAAGCG
GCAATGCGCAATGTGCATTGCCGGCGATTTTgtgagtttttttttttggctgaaagttttaatcaattatttattgtcacagTTGGACAAAAAG
GATCTGATGGTGGATGTATCTATTGGCCAGATGATTGTGTGGAACGATGAATGGGATTTAACAGACTGTTGG
ACTGGGGATGTCGTCATCAAAGAATGTGCCTCGGCATGTGTCTCCATATACACCAAGTCGAAAACCCGTGA
AGGATGGTTTTGGACAAgtgggctcaattgatcgtattttaactttttttcaaaaaattttcagGTGTCCTTATGGATTGTTCCGAGGC

Strain name and genotype: PHX5135 *folr-1(syb5135)*

-:deletion site
folr-1 exons are labeled with yellow and orange.

> *syb5135* (-1045bp)

agtcgttggcaaaatgtgggagaaataggtactagcctactgtgagcccatataattccaacagtatattttccagttgaaattctaagcgctttccaagggtgaaatagtatttttaaatt
gtcgtttgtgagtcatcataaatatatatgaaaatttgcttaggagagatgaaggtcacgcccatttgttatgagtcacaatcaaccgtactcgattttgtgcccctcttcttctctatttaaa
tgggatatctttgtttataacaatcatttttttaattccttgctgttctcaaacaaaattgccgataattatttttcagaa-
gctctacaaatcaactcgaggatgataccgaaacttatacttacttctcaactatttttaaatgtttcgaatattctttctttcctacggttcctatataataattatcttgtttttttattggtgcatt
ctttattttctaccgtttacagtattccttctacatattattatgaccaccctattagacttccactctacattagtattgtatctcgaacaccctctgagaacttgtatttcactcgttataagtttt
acaattccgattagtcttgcccagtaccgcctcattaattacagtattttaacaatgatttttttgcgttttggcaataattacacaatatcatttattttctattaaaggtggtgtagtagaattttt
attgctttattaggttaaaaattgtctgaaaacaccgaaaaaaaatgaatcaccgtcaaattttgggtatacatgtcaattatcttgcgttttcaacttcatttaggtattttaaagtcgatgg
gcggcgaggtttttaaattttttaaaccaaatctcgccgtccatcgaatttaaaatacctaaatgaagttgaaaacgcaagataattgacatgtatacccaaaatttgacggtgaaaaa
aattgtttagccgctgcgacattgacaagtcggtc

> wild type

agtcgttggcaaaatgtgggagaaataggtactagcctactgtgagcccatataattccaacagtatattttccagttgaaattctaagcgctttccaagggtgaaatagtatttttaaatt
gtcgtttgtgagtcatcataaatatatatgaaaatttgcttaggagagatgaaggtcacgcccatttgttatgagtcacaatcaaccgtactcgattttgtgcccctcttcttctctatttaaa
tgggatatctttgtttataacaatcatttttttaattccttgctgttctcaaacaaaattgccgataattatttttcagaaATGAGATTCAAACTTGTTTGTCTTTTTTT
TAAGTCTATTTGCTCTTCCGAGTGTGTCCCTCACATGGGACATGAGCAAGTGGATGCCAGACACCCGGACTC
ACTCGATGGTGAAGCGGCAATGCGCAATGTGCATTGCCGGCGATTTTgtgagtttttttttttggctgaaagttttaatcaattatttattgt
cacagTTGGACAAAAAGGATCTGATGGTGGATGTATCTATTGGCCAGATGATTGTGTGGAACGATGAATGGGA
TTTAACAGACTGTTGGACTGGGGATGTCGTCATCAAAGAATGTGCCTCGGCATGTGTCTCCATATACACCAA
GTCGAAAACCCGTGAAGGATGGTTTTGGACAAgtgggctcaattgatcgtattttaactttttttcaaaaaattttcagGTGTCCTTATGGA
TTGTTCCGAGGCATTGATCTGGTCATCACCCGATTTGTCTCTCAAACCGACGACAATGGTACTCTTAGACGG
AGTGTATGTTGCATCCAGAAAAGGCCACGATATCAAATATATATTCTCTACAAACAATAACAACAGCACAT
TGGACATCAGAGAGCATATATTTCTTTCATGGTTCCTGGAATCAGACAGAAAAGAACTACCTTCGGGACA

AAAGTCCTATTGGCTTCCATTGCCGTCATAATGATTTTTGTCGTTCTCGCATTATTAAAAATTGTGTACAAAG
AAGAGgtaataacactgatctcgaatttttttttttgtaaagttgaacatacaaaatacacctgttcaaacttctgggtgaagtcctatttaatttgaacattcttcagCGTAGCC
GAAAAAATAAAGATGATCGTGAGCCAGTGGTTAGCTACTGTCGTGACATTAAAGAGCATATTGCAAGAATG
GAACGCGGGCAAATTAAGCATGGGAAAGAGTTTAACAAGAACGTAGAGAATGTCCAAGACAATTTCACCG
ACAAGGATGATGCACACATGACGGACAATGAGTGTAATATGAACATCACTGATAATCAACAGCCTAAATAA
gctctacaaatcaactcgaggatgataccgaaacttatacttacttctcaactatttttaaatgtttcgaatattctttctttcctacggttcctatataataattatcttgtttttttattggtgcatt
ctttattttctaccgtttacagtattccttctacatattattatgaccaccctattagacttccactctacattagtattgtatctcgaacaccctctgagaacttgtatttcactcgttataagtttt
acaattccgattagtcttgcccagtaccgcctcattaattacagtattttaacaatgatttttttgcgttttggcaataattacacaatatcatttattttctattaaaggtggtgtagtagaattttt
attgctttattaggttaaaaattgtctgaaaacaccgaaaaaaaatgaatcaccgtcaaattttgggtatacatgtcaattatcttgcgttttcaacttcatttaggtattttaaagtcgatgg
gcggcgaggtttttaaatttttttaaaccaaatctcgccgtccatcgaatttaaaatacctaaatgaagttgaaaacgcaagataattgacatgtatacccaaaatttgacggtgaaaaa
aattgtttagccgctgcgacattgacaagtcggtc

Strain name and genotype: PHX4185 *folr-1(syb4185[folr-1::mNeonGreen])* X

Synonymous mutation is labeled in blue. *folr-1* exons are labeled with yellow and orange and mNeonGreen is labeled in green.

> *syb4185*

gcccatttgttatgagtcacaatcaaccgtactcgattttgtgcccctcttcttctctatttaaatgggatatctttgtttataacaatcatttttttaattccttgctgttctcaaacaaaattgccg
ataattatttttcagaaATGAGATTCAAACTTGTTTGTCTTTTTTTAAGTCTATTTGCTCTTCCGAGTGTGTCCCTCACAT
GGGACATGAGCAAGTGGATGCCAGACACCCGGACTCACTCGATGGTGAAGCGGCAATGCGCAATGTGCATT
GCCGGCGATTTTgtgagtttttttttttttggctgaaagttttaatcaattatttattgtcacagTTGGACAAAAAGGATCTGATGGTGGATGTAT
CTATTGGCCAGATGATTGTGTGGAACGATGAATGGGATTTAACAGACTGTTGGACTGGGGATGTCGTCATC
AAAGAATGTGCCTCGGCATGTGTCTCCATATACACCAAGTCGAAAACCCGTGAAGGATGGTTTTGGACAAgt
gggctcaattgatcgtattttaactttttttcaaaaaattttcagGTGTCCTTATGGATTGTTCCGAGGCATTGATCTGGTCATCACCCGAT
TTGTCTCTCAAACCGACGACAATGGTACTCTTAGACGGAGTGTATGTTGCATCCAGAAAAGGCCACGATATC
AAATATATATTCTCTACAAACAATAACAACAGCACATTGGACATCAGAGAGCATATATTTTCTTTCATGGTT
CCTGGAATCAGACAGAAAAGAACTACCTTCGGGACAAAAGTCCTATTGGCTTCCATTGCCGTCATAATGATT
TTTGTCGTTCTCGCATTATTAAAAATTGTGTACAAAGAAGAGgtaataacactgatctcgaatttttttttttgtaaagttgaacatacaaaatac
acctgttcaaacttctgggtgaagtcctatttaatttgaacattcttcagCGTAGCCGAAAAAATAAAGATGATCGTGAGCCAGTGGTTAGC
TACTGTCGTGACATTAAAGAGCATATTGCAAGAATGGAACGCGGGCAAATTAAGCATGGGAAAGAGTTTAA
CAAGAACGTAGAGAATGTCCAAGACAAT**TTTACT**GACAAGGATGATGCACACATGACGGACAATGAGTGT
AATATGAACATCACTGATAATCAACAGCCTAAAATGGTCTCCAAGGGAGAGGAGGACAACATGGCCTCCCT
CCCAGCCACCCACGAGCTCCACATCTTCGGATCCATCAACGGAGTCGACTTCGACATGGTCGGACAAGGAA
CCGGAAACCCAAACGACGGATACGAGGAGCTCAACCTCAAGTCCACCAAGgtaagtttaaacatatatatactaactaaccctgat
tatttaaattttcagGGAGACCTCCAATTCTCCCCATGGATCCTCGTCCCACACATCGGATACGGATTCCACCAATACC
TCCCATACCCAGACGGAATGTCCCCATTCCAAGCCGCCATGGTCGACGGATCCGGATACCAAGTCCACCGT
ACCATGCAATTCGAGGACGGAGCCTCCCTCACCGTCAACTACCGTTACACCTACGAGGGATCCCACATCAA
GgtaagtttaaacagttcggtactaactaaccatacatatttaaattttcagGGAGAGGCCCAAGTCAAGGGAACCGGATTCCCAGCCGACG
GACCAGTCATGACCAACTCCCTCACCGCCGCCGACTGGTGCCGTTCCAAGAAGACCTACCCAAACGACAAG
gtaagtttaaacatgatttactaactaactaatctgatttaaattttcagACCATCATCTCCACCTTCAAGTGGTCCTACACCACCGGAAACG
GAAAGCGTTACCGTTCCACCGCCCGTACCACCTACACCTTCGCCAAGCCAATGGCCGCCAACTACCTCAAG
AACCAACCAATGTACGTCTTCCGTAAGACCGAGCTCAAGCACTCCAAGACCGAGCTCAACTTCAAGGAGTG
GCAAAAGGCCTTCACCGACGTCATGGGAATGGACGAGCTCTACAAGTAAgctctacaaatcaactcgaggatgataccgaaactt
atacttacttctcaactatttttaaatgtttcgaatattctttctttcctacggttcctatataataattatcttgtttttttattggtgcattctttattttctaccgtttacagtattccttctacatattatt
atgaccaccctattagacttccactctacattagtattgtatctcgaacaccctctgagaacttgtatttcactcgttataagttttacaattccgattagtcttgcccagtaccgcctcatta
attacagtattttaacaatgatttttttgcgttttggcaataattacacaatatcatttattttctattaaaggtggtgtagtagaatttttattgctttattaggttaaaaattgtctgaaaacaccg
aaaaaaaatgaatcaccgtcaaattttgggtatacatgtcaattatcttgcgttttcaacttcatttaggtattttaaagtcgatgggcggcgaggtttttaaatttttttaaaccaaatctcgc
cgtccatcgaatttaaaatacctaaatgaagttgaaaacgcaagataattgacatgtatacccaaaatttgacggtgaaaaaaattgtttagccgctgcgacattgacaagtcggtcaa
atttcaaattttacctaatttaacgcaatttttttgagtcgtcataacttttttttgagaagttttcaaaaagtttcattatgaaattcggtgttttcagacaattttgagtctaataaagaaataaaaa
attcgactacaccatttttaatttcatacaccgaaggaattgaaacataatcagagaatacaaatcatttcactaagtcttttaaatttttttgaatagagtttcaaaaagctcgtttacatatta
aaatacgattttccttccagtgtctgtcgttttgtttcaaacatgcgggatatttttttcaacatgagaatagatcatcaaattgttttgcagacacaaaaacactttgctaaagtgaaactgtc
acattccatttacttttcctatcttatcccacgcaaat

Strain name and genotype: PHX7330 *rps-6(syb7330[rps-6::wrmScarlet])* I

Synonymous mutation is labeled in blue. *rps-6* exons are labeled with yellow and orange (only part of exon sequences included) and wrmScarlet is labeled in red.

> *syb7330*
GACCAAAGAGAGCCTCCAAGATCCGCAAGCTCTTCAACTTGACCAAGCACGACGATGTCACCAAATACGTC
ATCACCCACGACAAGACCTTCCCAGATGgtgagtttcttcaatattctctttaaatttagtttttaactgtctaaatttccagGAGTCACCAAGAC
CATCGCACCAAAGATCCAGCGTCTCATCACCCCAGCTCGTATCGCCAGAAAGAAGTACCTTCTCCGCCAGA
AGCGCAACCAGAAGATCAAGATGCGCGATGACGCCGCTGCCTACCACAAGCTCCTCGCCAAGTACTCCAAG
GAAGAGCACGACGCCAAGATCGCCCGCAGACGCTCTTCGGCTTCCCATCACTCCGAGAGCGAGGTCAAGAA
GACCAGCAAGAAGATGGTCAGCAAGGGAGAGGCAGTTATCAAGGAGTTCATGCGTTTCAAGGTCCACATG
GAGGGATCCATGAACGGACACGAGTTCGAGATCGAGGGAGAGGGAGAGGGACGTCCATACGAGGGAACCC
AAACCGCCAAGCTCAAGGTCACCAAGGGAGGACCACTCCCATTCTCCTGGGACATCCTCTCCCCACAATTC
ATGTACGGATCCCGTGCCTTCACCAAGCACCCAGCCGACATCCCAGACTACTACAAGCAATCCTTCCCAGA
GGGATTCAAGTGGGAGCGTGTCATGAACTTCGAGGACGGAGGAGCCGTCACCGTCACCCAAGACACCTCCC
TCGAGGACGGAACCCTCATCTACAAGGTCAAGCTCCGTGGAACCAACTTCCCACCAGACGGACCAGTCATG
CAAAAGAAGACCATGGGATGGGAGGCCTCCACCGAGCGTCTCTACCCAGAGGACGGAGTCCTCAAGGGAG
ACATCAAGATGGCCCTCCGTCTCAAGGACGGAGGACGTTACCTCGCCGACTTCAAGACCACCTACAAGGCC
AAGAAGCCAGTCCAAATGCCAGGAGCCTACAACGTCGACCGTAAGCTCGACATCACCTCCCACAACGAGGA
CTACACCGTCGTCGAGCAATACGAGCGTTCCGAGGGACGTCACTCCACCGGAGGAATGGACGAGCTCTACA
AGTAAatactgctgctcgttattgtttcctaatgaaattgttgttaaacttaaatcttttttttttttgttgttcactccattcgtctcaccaattatatttgcacccatttgtcgttgttaattttttttg
aatgcttactccgagtcctggcacgccactcagggtacttggtcgatccgttcttcgcctgcggggcggatagcaggcttcgcagctttcaaagcttttcggttctttgcttggctgat
agttgtatgactcaaccgaaggcgtctctcgtgtgacctttttataaagagtcgttccatctaaacgggagtcgtccatttttgtgttaattctgctaagataaagcgcgctcctgaaatta
tcttccaaagtgctgtgattctttttcaaggatacggtagaagtcttgc

Strain name and genotype: PHX4824 and PHX4825 *Is[Pfolr-1::folr-1::unc-54 3'UTR, Pmyo-2::gfp]* (*Pmyo-2::gfp* was used as a marker for transgenic animals)

Sequence of Ppd49.78(*Pfolr-1::folr-1::unc-54* 3'UTR) -plasmid

folr-1 promoter is labeled with green, *folr-1* exons are labeled with yellow and orange, part of the last *unc-54* exon is labeled with olive green and *unc-54* 3' UTR is labeled in purple.

ATGACCATGATTACGCC**AAGCTT**agtgttccctctcattagtattaatacaatgtgaaaattcttccattattttttgtgttcttcacatttttaaaattatattttattcgtt
cacgttttaataaaaatggaaagaaggagaatcaaaggattttagaggaaaaacatgtgatcgataagatgatcaagtaaccgttatatggttgggtgatcaactatgagaaagaaa
tttatcaagaggtaaatagcttataagtgctgcgaaggctaagcgggaaagtttaaccttttttttttaataaaaaaataatctagctatactgtttcaggagggaaaatacttatgtaaga
agctgttgttggaggggccacaagtacacagcaagctggaaaatctaggaaatcgggcttttctgacaagagaggaaaatgaaaatcaccgcaaatagtgttctggtacgtcctg
aggatttgtaggatttgcctttgttgaactgatttaagatagaattggttgagacgatcatcgttggaggagtatatcaagttaatttttttgaagaaacgtcgggagaagtatttcagcggt
ttctcaagttttttttgtcaagaatattgaactcggactgtagatctcgctttcataatccaaaacaagttatacggagatgttgtagagtttgaaataaagattggcattggtggagcggaa
tgttaaacagttgtctgcatttcagtagtgcagcattcgagaaaaattgatgtgagcattaaagttgagcttgttgtcgacaaatattcctagatccttgactgattccttctttgcaatcttg
ataccattgtcaaagtaatccatctttgggtttttttctgaagagtaggactgcggacttactttccaccagaaataattttcagttggtgcaccagtttggtactatgttgtttgatgtttcaa
ttgatttaccgttttctccaaatattttgagatcatccgcgaaggcggagatgtggacgtcgtttgggagcaatttcagaaaatgacttaaaccagttgcatatttttacgttgcccgacc
cggcagagctaagaaaatttttcaaaaacggcgaaacggagggttttagtttttttttttaattttagagataaattggtctatgatggacatgtttgcgagtcgttggcaaaatgtggga
gaaataggtactagcctactgtgagcccatataattccaacagtatattttccagttgaaattctaagcgctttccaagggtgaaatagtatttttaaattgtcgtttgtgagtcatcataaa
tatatatgaaaatttgcttaggagagatgaaggtcacgcccatttgttatgagtcacaatcaaccgtactcgattttgtgcccctcttcttctctatttaaatgggatatctttgtttataacaa
tcatttttttaattccttgctgttctcaaacaaaattgccgataattatttttcagaaATGAGATTCAAACTTGTTTGTCTTTTTTTAAGTCTATTTGCT
CTTCCGAGTGTGTCCCTCACATGGGACATGAGCAAGTGGATGCCAGACACCCGGACTCACTCGATGGTGAA
GCGGCAATGCGCAATGTGCATTGCCGGCGATTTTgtgagtttttttttttggctgaaagttttaatcaattatttattgtcacagTTGGACAAA
AAGGATCTGATGGTGGATGTATCTATTGGCCAGATGATTGTGTGGAACGATGAATGGGATTTAACAGACTG
TTGGACTGGGGATGTCGTCATCAAAGAATGTGCCTCGGCATGTGTCTCCATATACACCAAGTCGAAAACCC
GTGAAGGATGGTTTTGGACAAgtgggctcaattgatcgtattttaactttttttcaaaaaaattttcagGTGTCCTTATGGATTGTTCCGAGGC
ATTGATCTGGTCATCACCCGATTTGTCTCTCAAACCGACGACAATGGTACTCTTAGACGGAGTGTATGTTGC

ATCCAGAAAAGGCCACGATATCAAATATATATTCTCTACAAACAATAACAACAGCACATTGGACATCAGAG
AGCATATATTTCTTTCATGGTTCCTGGAATCAGACAGAAAAGAACTACCTTCGGGACAAAAGTCCTATTGG
CTTCCATTGCCGTCATAATGATTTTTGTCGTTCTCGCATTATTAAAAATTGTGTACAAAGAAGAGgtaataacactga
tctcgaattttttttttgtaaagttgaacatacaaaatacacctgttcaaacttctgggtgaagtcctatttaatttgaacattcttcagCGTAGCCGAAAAAAATAAAGA
TGATCGTGAGCCAGTGGTTAGCTACTGTCGTGACATTAAAGAGCATATTGCAAGAATGGAACGCGGGCAAA
TTAAGCATGGGAAAGAGTTTAACAAGAACGTAGAGAATGTCCAAGACAATTTCACCGACAAGGATGATGC
ACACATGACGGACAATGAGTGTAATATGAACATCACTGATAATCAACAGCCTAAATAAGAGCTCCGCATCG
GCCGCTGTCATCAGATCGCCATCTCGCGCCCGTGCCTCTGACTTCTAAGTCCAATTACTCTTCAACATCCCTA
CATGCTCTTTCTCCCTGTGCTCCCACCCCCTATTTTTGTTATTATCAAAAAACTTCTCTTAATTTCTTTGTTTTT
TAGCTTCTTTTAAGTCACCTCTAACAATGAAATTGTGTAGATTCAAAAATAGAATTAATTCGTAATAAAAAG
TCGAAAAAAATTGTGCTCCCTCCCCCCATTAATAATAATTCTATCCCAAAATCTACACAATGTTCTGTGTAC
ACTTCTTATGTTTTTACTTCTGATAAATTTTTTTGAAACATCATAGAAAAAACCGCACACAAAATACCTTAT
CATATGTTACGTTTCAGTTTATGACCGCAATTTTTATTTCTTCGCACGTCTGGGCCTCTCATGACGTCAAATC
ATGCTCATCGTGAAAAAGTTTTGGAGTATTTTTGGAATTTTTCAATCAAGTGAAAGTTTATGAAATTAATTTT
CCTGCTTTTGCTTTTTGGGGTTTCCCCTATTGTTTGTCAAGATTTCGAGGACGGCGTTTTTCTTGCTAAAATCA
CAAGTATTGATGAGCACGATGCAAGAAAGATCGGAAGAAGGTTTGGGTTTGAGGCTCAGTGGAAGGTGAG
TAGAAGTTGATAATTTGAAAGTGGAGTAGTGTCTATGGGGTTTTTGCCTTAAATGACAGAATACATTCCCAA
TATACCAAACATAACTGTTTCCTACTAGTCGGCCGTACGGGCCCTTTCGTCTCGCGCGTTTCGGTGATGACG
GTGAAAACCTCTGACACATGCAGCTCCCGGAGACGGTCACAGCTTGTCTGTAAGCGGATGCCGGGAGCAGA
CAAGCCCGTCAGGGCGCGTCAGCGGGTGTTGGCGGGTGTCGGGGCTGGCTTAACTATGCGGCATCAGAGCA
GATTGTACTGAGAGTGCACCATATGCGGTGTGAAATACCGCACAGATGCGTAAGGAGAAAATACCGCATCA
GGCGGCCTTAAGGGCCTCGTGATACGCCTATTTTTATAGGTTAATGTCATGATAATAATGGTTTCTTAGACG
TCAGGTGGCACTTTTCGGGGAAATGTGCGCGGAACCCCTATTTGTTTATTTTTCTAAATACATTCAAATATGT
ATCCGCTCATGAGACAATAACCCTGATAAATGCTTCAATAATATTGAAAAAGGAAGAGTATGAGTATTCAA
CATTTCCGTGTCGCCCTTATTCCCTTTTTTGCGGCATTTTGCCTTCCTGTTTTTGCTCACCCAGAAACGCTGGT
GAAAGTAAAAGATGCTGAAGATCAGTTGGGTGCACGAGTGGGTTACATCGAACTGGATCTCAACAGCGGTA
AGATCCTTGAGAGTTTTCGCCCCGAAGAACGTTTTCCAATGATGAGCACTTTTAAAGTTCTGCTATGTGGCG
CGGTATTATCCCGTATTGACGCCGGGCAAGAGCAACTCGGTCGCCGCATACACTATTCTCAGAATGACTTGG
TTGAGTACTCACCAGTCACAGAAAAGCATCTTACGGATGGCATGACAGTAAGAGAATTATGCAGTGCTGCC
ATAACCATGAGTGATAACACTGCGGCCAACTTACTTCTGACAACGATCGGAGGACCGAAGGAGCTAACCGC
TTTTTTGCACAACATGGGGGATCATGTAACTCGCCTTGATCGTTGGGAACCGGAGCTGAATGAAGCCATACC
AAACGACGAGCGTGACACCACGATGCCTGTAGCAATGGCAACAACGTTGCGCAAACTATTAACTGGCGAAC
TACTTACTCTAGCTTCCCGGCAACAATTAATAGACTGGATGGAGGCGGATAAAGTTGCAGGACCACTTCTGC
GCTCGGCCCTTCCGGCTGGCTGGTTTATTGCTGATAAATCTGGAGCCGGTGAGCGTGGGTCTCGCGGTATCA
TTGCAGCACTGGGGCCAGATGGTAAGCCCTCCCGTATCGTAGTTATCTACACGACGGGGAGTCAGGCAACT
ATGGATGAACGAAATAGACAGATCGCTGAGATAGGTGCCTCACTGATTAAGCATTGGTAACTGTCAGACCA
AGTTTACTCATATATACTTTAGATTGATTTAAAACTTCATTTTTAATTTAAAAGGATCTAGGTGAAGATCCTT
TTTGATAATCTCATGACCAAAATCCCTTAACGTGAGTTTTCGTTCCACTGAGCGTCAGACCCCGTAGAAAAG
ATCAAAGGATCTTCTTGAGATCCTTTTTTTCTGCGCGTAATCTGCTGCTTGCAAACAAAAAAACCACCGCTA
CCAGCGGTGGTTTGTTTGCCGGATCAAGAGCTACCAACTCTTTTTCCGAAGGTAACTGGCTTCAGCAGAGCG
CAGATACCAAATACTGTCCTTCTAGTGTAGCCGTAGTTAGGCCACCACTTCAAGAACTCTGTAGCACCGCCT
ACATACCTCGCTCTGCTAATCCTGTTACCAGTGGCTGCTGCCAGTGGCGATAAGTCGTGTCTTACCGGGTTG
GACTCAAGACGATAGTTACCGGATAAGGCGCAGCGGTCGGGCTGAACGGGGGGTTCGTGCACACAGCCCA
GCTTGGAGCGAACGACCTACACCGAACTGAGATACCTACAGCGTGAGCATTGAGAAAGCGCCACGCTTCCC
GAAGGGAGAAAGGCGGACAGGTATCCGGTAAGCGGCAGGGTCGGAACAGGAGAGCGCACGAGGGAGCTT
CCAGGGGGAAACGCCTGGTATCTTTATAGTCCTGTCGGGTTTCGCCACCTCTGACTTGAGCGTCGATTTTTGT
GATGCTCGTCAGGGGGGCGGAGCCTATGGAAAAACGCCAGCAACGCGGCCTTTTTACGGTTCCTGGCCTTTT
GCTGGCCTTTTGCTCACATGTTCTTTCCTGCGTTATCCCCTGATTCTGTGGATAACCGTATTACCGCCTTTGA
GTGAGCTGATACCGCTCGCCGCAGCCGAACGACCGAGCGCAGCGAGTCAGTGAGCGAGGAAGCGGAAGAG
CGCCCAATACGCAAACCGCCTCTCCCCGCGCGTTGGCCGATTCATTAATGCAGCTGGCACGACAGGTTTCCC
GACTGGAAAGCGGGCAGTGAGCGCAACGCAATTAATGTGAGTTAGCTCACTCATTAGGCACCCCAGGCTTT
ACACTTTATGCTTCCGGCTCGTATGTTGTGTGGAATTGTGAGCGGATAACAATTTCACACAGGAAACAGCT
