# Supplementary figures and images for: Folate receptor overexpression induces toxicity in a diet-dependent manner in C. elegans
Source: Sci Rep. 2024 Jan 11;14:1066. doi: 10.1038/s41598-024-51700-9 (PMC10784478; doi:10.1038/s41598-024-51700-9)

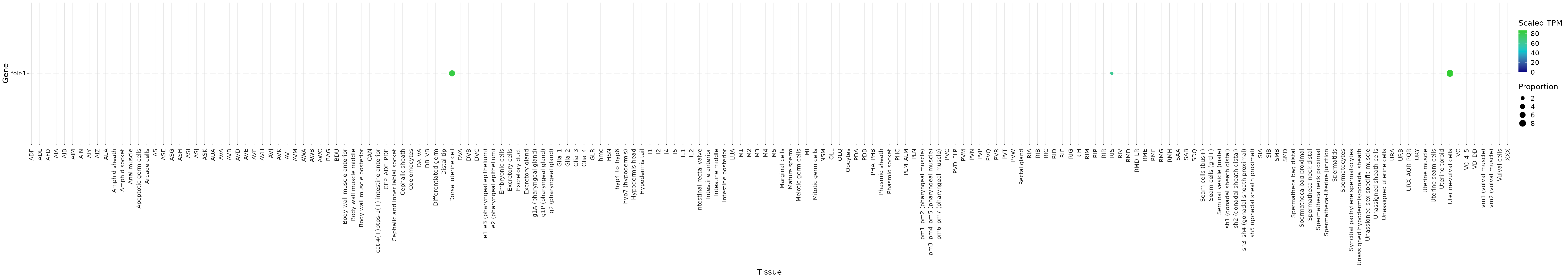

Supplement: Supplementary file 2 — Supplementary Figure S2. [file 41598_2024_51700_MOESM2_ESM.png]

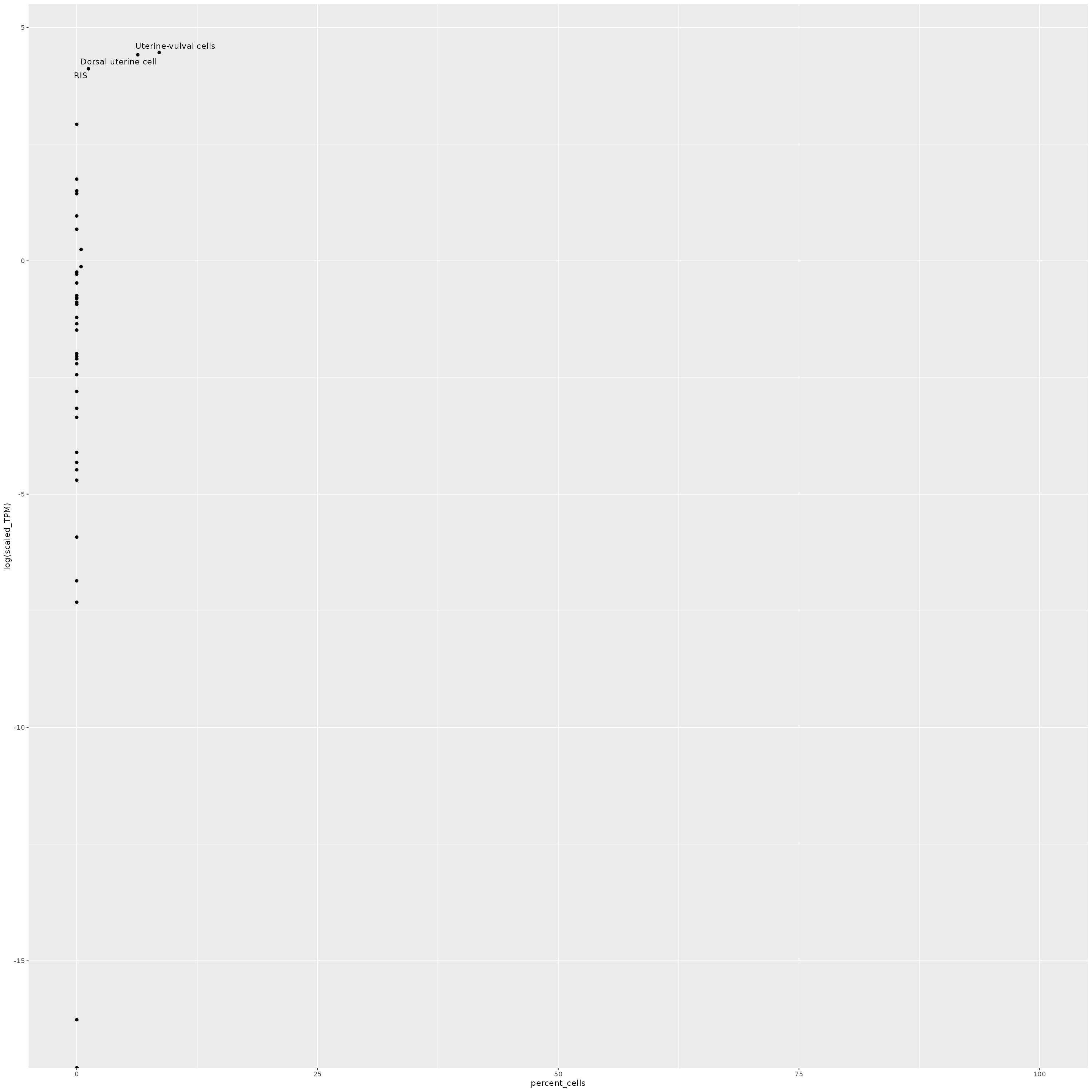

Supplement: Supplementary file 3 — Supplementary Figure S3. [file 41598_2024_51700_MOESM3_ESM.png]
